# Voxelwise Principal Component Analysis of Dynamic [S-Methyl-^11^C]Methionine PET Data in Glioma Patients

**DOI:** 10.3390/cancers13102342

**Published:** 2021-05-12

**Authors:** Corentin Martens, Olivier Debeir, Christine Decaestecker, Thierry Metens, Laetitia Lebrun, Gil Leurquin-Sterk, Nicola Trotta, Serge Goldman, Gaetan Van Simaeys

**Affiliations:** 1 Department of Nuclear Medicine, Hôpital Erasme, Université libre de Bruxelles, Route de Lennik 808, 1070 Brussels, Belgium; gil.leurquin-sterk@erasme.ulb.ac.be (G.L.-S.); nicola.trotta@erasme.ulb.ac.be (N.T.); sgoldman@ulb.ac.be (S.G.); gaetan.vansimaeys@ulb.ac.be (G.V.S.); 2 Laboratory of Image Synthesis and Analysis (LISA), École Polytechnique de Bruxelles, Université libre de Bruxelles, Avenue Franklin Roosevelt 50, 1050 Brussels, Belgium; odebeir@ulb.ac.be (O.D.); cdecaes@ulb.ac.be (C.D.); thierry.metens@erasme.ulb.ac.be (T.M.); 3 Department of Radiology, Hôpital Erasme, Université libre de Bruxelles, Route de Lennik 808, 1070 Brussels, Belgium; 4 Department of Pathology, Hôpital Erasme, Université libre de Bruxelles, Route de Lennik 808, 1070 Brussels, Belgium; laetitia.lebrun@erasme.ulb.ac.be

**Keywords:** [^11^C]MET PET, dynamic PET, glioma, pharmacokinetic modeling, principal component analysis, tumor heterogeneity

## Abstract

**Simple Summary:**

Recent works on dynamic amino acid positron emission tomography (PET) imaging of gliomas have highlighted characteristic behaviors of time-activity curves (TACs) extracted from the whole tumor w.r.t. the grade, genotype, and outcome. However, gliomas are known to be highly heterogeneous tumors. Here, we aim at highlighting similar dynamic behaviors at the voxel level within the tumor volume in [S-methyl-^11^C]methionine PET data of 33 glioma patients using principal component analysis (PCA). The PCA model was derived from TACs of 20 patients and subsequently applied to 13 other patients in whom our approach was shown to outperform classical pharmacokinetic modeling to this end. Our parameter-free approach provides additional parametric maps from dynamic methionine PET scans with little modification of the routine protocol and no arterial sampling. This early methodological work paves the way for various clinical studies on glioma heterogeneity with applications for treatment planning and response evaluation.

**Abstract:**

Recent works have demonstrated the added value of dynamic amino acid positron emission tomography (PET) for glioma grading and genotyping, biopsy targeting, and recurrence diagnosis. However, most of these studies are based on hand-crafted qualitative or semi-quantitative features extracted from the mean time activity curve within predefined volumes. Voxelwise dynamic PET data analysis could instead provide a better insight into intra-tumor heterogeneity of gliomas. In this work, we investigate the ability of principal component analysis (PCA) to extract relevant quantitative features from a large number of motion-corrected [S-methyl-^11^C]methionine ([^11^C]MET) PET frames. We first demonstrate the robustness of our methodology to noise by means of numerical simulations. We then build a PCA model from dynamic [^11^C]MET acquisitions of 20 glioma patients. In a distinct cohort of 13 glioma patients, we compare the parametric maps derived from our PCA model to these provided by the classical one-compartment pharmacokinetic model (1TCM). We show that our PCA model outperforms the 1TCM to distinguish characteristic dynamic uptake behaviors within the tumor while being less computationally expensive and not requiring arterial sampling. Such methodology could be valuable to assess the tumor aggressiveness locally with applications for treatment planning and response evaluation. This work further supports the added value of dynamic over static [^11^C]MET PET in gliomas.

## 1. Introduction

Gliomas are the most common primary brain tumors and are associated with poor prognosis. Glioma diagnosis and follow-up usually rely on magnetic resonance imaging (MRI), although the addition of positron emission tomography (PET) with radio-labeled amino acids such as [S-methyl-^11^C]methionine ([^11^C]MET) has been shown to provide complementary information for tumor delineation [1] and characterization [2,3], as well as for biopsy [4,5,6] and therapy [7] planning. Whereas clinical amino acid PET imaging of gliomas is almost exclusively based on static acquisitions, the added value of dynamic PET acquisitions has been demonstrated for tumor grading and genotyping [8,9,10,11,12,13], biopsy targeting [9], and recurrence diagnosis [14]. Aside from a longer acquisition time, the main limitation of dynamic PET imaging lies in the difficulty of extracting robust and clinically relevant features from noisy time-activity curves (TACs).

Previous works on dynamic PET imaging of gliomas with O-(2-[^18^F]fluoroethyl)-L-tyrosine ([^18^F]FET)—another amino acid PET tracer equivalent to [^11^C]MET [15]—have highlighted differences in uptake dynamics between high-grade gliomas (HGGs) and low-grade gliomas (LGGs) by visually labeling mean tumor TACs as ‘increasing’ or ‘decreasing’. It has been shown that a fast increasing then progressively decreasing mean TAC is characteristic of HGGs whereas a slowly increasing mean TAC is rather observed in LGGs [8,10], and that foci with a decreasing TAC should be taken into account for surgery guidance [9]. These interesting findings however have some methodological limitations since TAC labeling does not allow continuous quantification of the dynamic behavior and voxelwise extension might become challenging for large amounts of noisy data.

Recently, time-to-peak (TTP) has been investigated as a dynamic feature of interest for quantitative characterization of the mean TAC in gliomas, with promising results for glioma grading [10,11,12,13,16] and recurrence diagnosis [14] in dynamic [^18^F]FET PET. TTP has the advantage of being easily computed and reflects to some extent the ‘increasing’ or ‘decreasing’ behavior of the TACs. However, this parameter is highly sensitive to data noise and depends on the PET reconstruction framing, hence TTP values lie in a discrete range of arbitrarily chosen times.

Pharmacokinetic (PK) modeling is the gold standard method for dynamic PET data analysis. PK modeling relies on compartmental models whose kinetic parameters are estimated from the observed TACs given an arterial input function (AIF) (i.e., the TAC of arterial blood used as an input for the model). This method has the great advantage of providing biologically interpretable kinetic parameters and has been previously used for glioma delineation in 2-deoxy-2-[^18^F]fluoro-D-glucose [17] as well as for glioma genotyping in [^18^F]FET PET [11,18]. However, direct kinetic parameters fitting has two major limitations. First, it requires the user to provide an AIF, which can be either measured from arterial blood samples or extracted from large vessels appearing in the image. Nevertheless, arterial sampling is an invasive procedure and is inconvenient in clinical practice. Image-derived input functions (IDIFs), on the other hand, are affected by partial volume effects inherent to PET imaging and by blood tracer metabolites, with direct impact on the kinetic parameters estimation. Second, kinetic parameters fitting is a computationally expensive process and its voxelwise extension may result in substantially long computation times with increased impact of data noise. Alternative methods have been proposed for PK analysis of dynamic PET data such as graphical analysis or reference tissue models, the latter not requiring an AIF. However, these methods generally provide only macro or relative kinetic parameters. A comparison of commonly used PK analysis methods for parametric map extraction from dynamic [^18^F]FET PET data in diffuse gliomas has been performed recently by Koopman and colleagues [19].

Principal component analysis (PCA) is a commonly used unsupervised multivariate analysis technique aiming at reducing high dimensional data space into a reduced number of components that best explain the observed data variance. The use of PCA for dynamic PET data analysis has been intensively studied by means of simulations, though surprisingly few clinical applications have seemed to emerge from these works. One major limitation of this technique is its limited ability to separate signal from noise for high noise levels or non-Gaussian noise distributions. The impact of noise level and distribution on PCA of dynamic PET images has been previously studied using synthetic imaging datasets by Pedersen and colleagues [20] and Šámal and colleagues [21].

Most of the published dynamic PET studies in gliomas, including those mentioned above, are only concerned with the mean TAC inside one or several pre-delineated volume(s) of interest (VOI(s)). However, gliomas are known to be highly heterogeneous tumors that may comprise multiple subregions with varying genotype, proliferation potential, aggressiveness, hypoxia level, and treatment-resistance abilities. Though computationally more expensive and more prone to noise, voxelwise analysis of dynamic PET data could instead provide a valuable intra-tumor insight. Voxelwise extensions of TTP analysis [16] and PK modeling [18] have been recently proposed but are still prone to the same limitations as their regionwise counterpart, with increased impact of noise and stability issues reported for PK modeling [18].

Besides, most dynamic PET studies rely on a limited number of non-uniformly sampled and non-overlapping frames with variable length and no prior motion correction. However, uniform TAC sampling with a large number of short frames could potentially benefit the dynamic analysis by providing a higher temporal resolution and removing the need to select an arbitrary irregular framing, at the expense of data noise. In this sense, reconstruction of overlapping frames leads to a good comprise between temporal resolution and count statistics and has been previously used for preclinical cardiac PET imaging [22]. Having access to high dimensional data—even noisy—with highly correlated variables is also particularly suitable for PCA.

In this work, we investigate the ability of PCA to extract meaningful dynamic features from a large number of uniformly sampled and motion-corrected [^11^C]MET PET frames in glioma patients. The emphasis is placed on the ability to quantify at the voxel level of the ‘increasing’ versus ‘decreasing’ behavior of TACs previously reported by Pöpperl and colleagues for the whole metabolic tumor [8] since it is expected to be related to the local aggressiveness of the tumor. To this extent, we first assess the robustness of the proposed methodology to noise by means of realistic numerical simulations. We then compare the derived parametric maps to these obtained from classical voxelwise PK analysis. We conclude that PCA outperforms PK modeling in discriminating the characteristic dynamic behaviors reported previously while not requiring arterial sampling. Our results also support the added value of dynamic over static analysis of [^11^C]MET PET data in gliomas, as previously demonstrated for [^18^F]FET.

## 2. Materials and Methods

### 2.1. Image Acquisition and Reconstruction

In total, 33 glioma patients (20 males, 28 surgically treated, of median age 55 year) admitted to our institution for a diagnosis or follow-up [^11^C]MET PET scan were enrolled in this study. Patient’s clinical data, 2016 WHO classification, and undergone treatments at imaging time are provided for each lesion in Table A1. The patient cohort was further split into PCA model-building (n=20, patients 1 to 20 in Table A1) and evaluation (n=13, patients 21 to 33 in Table A1) sets (see Section 2.8). All patients underwent a 30 min and 30 s PET acquisition started 30 s before the intravenous injection of 287–555 MBq of [^11^C]MET. All acquisitions were performed on a Vereos digital PET-CT scanner (Philips Healthcare, Best, The Netherlands) with an axial and trans-axial resolution of 4.1 mm at 1 cm from the field-of-view center. In total, 906 overlapping frames of 20 s, spaced by 2 s with a voxel size of 2 mm × 2 mm × 2 mm were reconstructed from the LIST files using time-of-flight ordered subset expectation maximization (TOF-OSEM, 10 subsets and 3 iterations) with computed tomography-based attenuation correction (CTAC). No post-reconstruction filter was applied to the dynamic frames. A routine static PET image (20–27 min post-injection (p.i.)) was also reconstructed for each patient (2 mm × 2 mm × 2 mm—CTAC TOF-OSEM, 15 subsets and 3 iterations—no filter).

### 2.2. Motion Correction

A systematic and progressive drift of the patients’ head throughout the acquisition was observed in our dataset, imputed to the low stiffness of the scanner head support. Although motion between two consecutive frames is almost unnoticeable, it turned out that the patients’ head had sunk of up to 1.5 mm over 30 min of acquisition. Such movement substantially impacts TACs at the voxel level and had to be corrected prior to the analysis. Since frame-by-frame registration is not suitable for short frames with a low signal-to-noise ratio (SNR), the following approach was used: Frames 36 to 906 (i.e., 40 s to 30 min p.i.) were grouped into consecutive blocks of 31 frames spaced by 30 frames. The mean frame of each block was computed, referred to as a ‘long frame.’ The last long frame was used as a reference for rigid registration of the other long frames by mutual information maximization [23]. Each computed rigid transform, expressed as a versor, was assigned to the mid-frame time point of the corresponding long frame. For each short frame, a rigid transform at mid-frame time was finally linearly interpolated from the known long frame transforms, which can be trivially performed when expressing transforms as versors. All registrations were performed in Python using SimpleITK [24].

### 2.3. Image-Derived Input Function Extraction

A blood input function was extracted from each registered volume for the pharmacokinetic analyses. A vascular image (0–120 s p.i.) was first computed by averaging registered frames 16 to 66. Two regions of interest (ROIs) covering the petrous (horizontal) segment of each internal carotid artery was then manually drawn on the early frame. The blood input function was finally obtained by averaging the TACs of the 10 brightest voxels on the vascular image within both ROIs. Brighter voxels are indeed considered to be the less impacted by partial volume effect (PVE), as suggested previously [25,26]. An estimation of the spill-out effects introduced was performed both analytically and by means of numerical simulations for realistic internal carotid arteries dimensions and scanner resolution (see Appendix A.2 and Appendix B.1). The estimated spill-out coefficient of 0.51 was used to correct the underestimated image-derived blood input functions.

### 2.4. Time-Activity Curves Extraction and Registration

All TACs within the brain region (excluding skull and peripheral cerebrospinal fluid) were extracted from each registered dynamic volume. For each patient, a brain ROI was first computed as follows: A late frame (20–30 min p.i.) was computed by averaging registered frames 616 to 906. The patient’s most recent T2 FLAIR MR image (no more than one month apart) was rigidly registered to the late frame by mutual information maximization [23]. The brain volume was then segmented on the registered T2-FLAIR image using a combination of thresholdings and morphological operations, available in an in-house C++ software based on VTK [27] and ITK [28]. All TACs within the brain ROI were spatially smoothed for noise reduction by averaging within a 3 vox × 3 vox × 3 vox neighborhood. For inter-patient normalization purpose, all smoothed TACs were converted to SUV units and temporally registered to account for inter-patient injection delay. The injection delay was computed for each patient by cross-correlation maximization between the patient’s IDIF and the most delayed IDIF observed, used as a reference (patient 17 in Table A1). In total, 6,420,534 TACs were extracted from the 30 min and 30 s [^11^C]MET PET dynamic scans of our 33 glioma patients.

### 2.5. Biological Tumor Volume Delineation

For each lesion, the biological tumor volume (BTV) was delineated on the average late frame (20–30 min p.i.) using a threshold of 1.6 times the normal brain uptake [29], computed as the mean SUV within a spherical ROI with radius 1 cm placed in the contralateral hemisphere symmetrically to the lesion location with regard to the falx cerebri. Basal nuclei were manually removed when erroneously included in the BTV. All segmentations were reviewed and approved by an experienced nuclear medicine physician.

### 2.6. Pharmacokinetic Modeling

**Figure 1:**
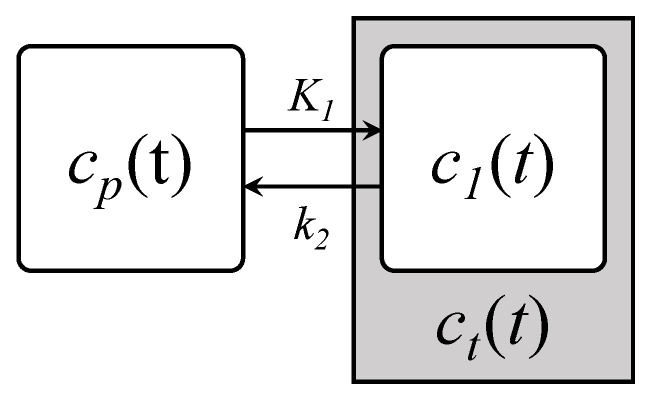
One-tissue compartment model (1TCM) describing the tracer transport from blood to tissues. $c_{p}\left(t\right)$ and $c_{1}\left(t\right)$ are, respectively, the tracer concentration in the blood and tissue compartment and $K_{1}$ and $k_{2}$ are the associated transport rate constants.

For short (30 min in this work) dynamic brain [^11^C]MET PET scans, the observed time variations in the voxel activity concentrations are mainly attributed to the tracer transport rather than protein synthesis [30]. The simplest compartmental model describing the transport of the tracer from blood to tissues is the one-tissue compartment model (1TCM) illustrated in Figure 1.

The transport processes involved are classically modeled by first-order kinetics, yielding:

(1) $$\frac{dc_{1}\left(t\right)}{dt}=K_{1}\;c_{p}\left(t\right)-k_{2}\;c_{1}\left(t\right),$$

where $c_{p}\left(t\right)$ and $c_{1}\left(t\right)$ are, respectively, the tracer activity concentration of the plasma and the tissue compartment at time *t*, and $K_{1}$ and $k_{2}$ are the transport rate constants from plasma to tissues and conversely. Calculating the unilateral Laplace transform of Equation (1) and considering zero initial concentration in both compartments leads to the expression of the transfer function $H_{1}\left(s\right)$ of the 1TCM:

(2) $$s\;C_{1}\left(s\right)=K_{1}\;C_{p}\left(s\right)-k_{2}\;C_{1}\left(s\right)\;\Leftrightarrow \;H_{1}\left(s\right)=\frac{C_{1}\left(s\right)}{C_{p}\left(s\right)}=\frac{K_{1}}{s+k_{2}},$$

where $C_{p}\left(s\right)$ and $C_{1}\left(s\right)$ are the respective Laplace transforms of $c_{p}\left(t\right)$ and $c_{1}\left(t\right)$ for Laplace variable *s*. In this work, we propose the following change of variables:

(3) $$G=\frac{K_{1}}{k_{2}},\;\tau =\frac{1}{k_{2}}\;\Leftrightarrow \;H_{1}\left(s\right)=\frac{G}{\tau \;s+1}.$$

This transformation is motivated by linear time-invariant system theory where *G* and τ are commonly referred to as the system static gain and time constant. From a physiological point a view, *G* is also referred to as the tracer total volume of distribution and τ as the inverse washout coefficient. *G* thus reflects the steady-state tissue to plasma uptake ratio (i.e., the asymptotic uptake value for a unit step plasma input function) whereas τ describes the uptake dynamics off-steady-state (i.e., the shape of the tissue TAC) and is expected to capture the sought-after ‘increasing’ or ‘decreasing’ behavior of tissue TACs. For the sake of clarity, the $K_{1}/k_{2}$ and $1/k_{2}$ notations will be used hereafter in place of *G* and τ.

Since a certain fraction of blood vessels is comprised within a single PET voxel (around 5% in healthy brain tissues), the whole voxel TAC is given by:

(4) $$c_{v}\left(t\right)=\alpha \;c_{b}\left(t\right)+\left(1-\alpha \right)\;c_{t}\left(t\right),$$

where α is the vascular fraction and $c_{v}\left(t\right)$, $c_{b}\left(t\right)$, and $c_{t}\left(t\right)$ are, respectively, the voxel, blood, and tissue total activity concentration, with $c_{t}\left(t\right)=c_{1}\left(t\right)$ for the 1TCM. Furthermore, $c_{b}$ can be expressed by:

(5) $$c_{b}\left(t\right)=h\;c_{e}\left(t\right)+\left(1-h\right)\;c_{p_{\mathrm{tot}}}\left(t\right),$$

where *h* is the haematocrit (i.e., the erythrocyte volume fraction of blood), $c_{e}\left(t\right)$ is the erythrocyte activity concentration, and $c_{p_{\mathrm{tot}}}\left(t\right)$ is the total plasma activity concentration attributed to the tracer and its metabolites. Since no blood samples were taken from the imaged patients and no population data were available for the erythrocyte uptake of [^11^C]MET, $c_{e}\left(t\right)$ was neglected in first approximation. The haematocrit *h* was set to its average population value of 0.45. The tracer plasma activity $c_{p}\left(t\right)$ was computed from the total plasma activity $c_{p_{\mathrm{tot}}}\left(t\right)$ using the time-dependent parent fraction f(t):

(6) $$c_{p}\left(t\right)=f\left(t\right)\;c_{p_{\mathrm{tot}}}\left(t\right).$$

**Figure 2:**
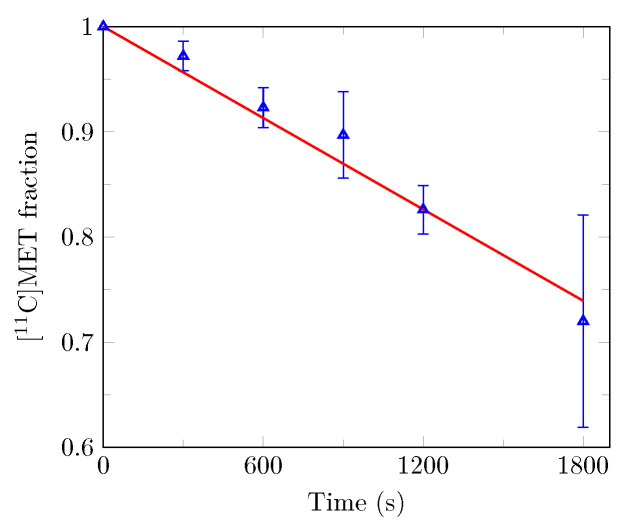
Time-dependent free [^11^C]MET plasma fraction used for metabolite correction. Population data points represented by blue triangles with error bars (mean ± standard deviation) were obtained by Sato and colleagues from 18 glioma patients [31]. The least-squares fitted linear approximation is plotted in red.

In this work, f(t) was linearly interpolated from population data points reported by Sato and colleagues [31] and depicted in Figure 2.

Taking into account the amount of time required for the tracer to flow from the carotid arteries—where the blood input function is extracted—to the voxel location, the voxel TAC is finally given by:

(7) $$c_{v}\left(t\right)=\alpha \;c_{b}\left(t-d\right)+\left(1-\alpha \right)\;h_{1}\left(t\right)*\left(\frac{f(t-d)}{1-h}\;c_{b}\left(t-d\right)\right),$$

where *d* is the system delay and $h_{1}$ is the inverse Laplace transform of $H_{1}$.

The 1TCM kinetic parameter $K_{1}$ and $k_{2}$ as well as the vascular fraction α and the delay *d* were individually estimated for each voxel TAC in our dataset (33 patients, 6,420,534 curves) by least-squares transfer function fitting using the SciPy’s ‘optimize’ and ‘signal’ modules [32] in Python. The added value of overlapping frames over adjacent frames for 1TCM kinetic parameters fitting was investigated by means of numerical simulations (see Appendix A.4 and Appendix B.2).

### 2.7. Numerical Simulations

Numerical simulations were conducted to assess the impact of data noise on the ability of PCA to capture characteristic TAC behaviors. Synthetic time activity curves were generated using the 1TCM and the tri-exponential blood input function model proposed by Feng and colleagues [33]:

(8) $$c_{b}\left(t\right)=\left(A_{1}\;t-A_{2}-A_{3}\right)\;e^{-\lambda_{1}t}+A_{2}\;e^{-\lambda_{2}t}+A_{3}\;e^{-\lambda_{3}t}.$$

To be able to compute an analytical solution for the tissue tracer activity, a linear approximation of the time dependent parent fraction f(t)=mt+p was built, leading to the following expression for the plasma input function:

(9) $$c_{p}\left(t\right)=\frac{m\;t+p}{1-h}\left(\left(A_{1}\;t-A_{2}-A_{3}\right)\;e^{-\lambda_{1}t}+A_{2}\;e^{-\lambda_{2}t}+A_{3}\;e^{-\lambda_{3}t}\right).$$

The value of *p* was fixed such that free [^11^C]MET plasma fraction is equal to 1 at injection time and the value of *m* was least-squares fitted on the experimental data of Sato and colleagues [31]. The resulting linear approximation is depicted in Figure 2 (red line).

Taking into account the carotid-to-voxel delay *d*, an analytical expression of the 1TCM tissue tracer activity $c_{t}\left(t\right)$ for t≥d and the plasma input function in Equation (9) is derived in Equation (A16) (see Appendix A.3). An analytical expression for the mean voxel activity concentration $c_{v}^{t_{s}\to t_{e}}$ for a frame starting at time $t_{s}$ and ending at time $t_{e}$ is then derived in Equations (A17)–(A19).

For each simulated frame $c_{v}^{t_{s}\to t_{e}}$ computed using Equation (A17), synthetic noise was generated using the model proposed by Logan and colleagues [34]:

(10) $$c_{v,\mathrm{noisy}}^{t_{s}\to t_{e}}=c_{v}^{t_{s}\to t_{e}}+\beta \;\sqrt{\frac{c_{v}^{t_{s}\to t_{e}}}{\left(t_{e}-t_{s}\right)\;e^{-\lambda \frac{t_{e}-t_{s}}{2}}}}\;G\left(0,1\right),$$

where $c_{v,\mathrm{noisy}}^{t_{s}\to t_{e}}$ is the noisy mean voxel tracer activity concentration for a frame starting at time $t_{s}$ and ending at time $t_{e}$, β is a scaling factor, λ is the isotope decay constant, and G(0,1) is a random number generated from a Gaussian distribution with mean 0 and standard deviation 1.

Synthetic TACs were generated using Equations (A17)–(A19) from the 1TCM kinetic parameter values previously fitted on each real TAC in our dataset (33 patients, 6,420,534 TACs). The input function parameter values used in Equation (8) were least-squares fitted on the corresponding patient’s IDIF using SciPy’s ‘optimize’ and ‘signal’ modules [32] in Python. The generated synthetic dataset is thus similar to our real dataset but has known ground truth signal. Synthetic noise was added to simulated TACs using Equation (10) for increasing noise levels β ranging from 0.25 to 2.0 with step 0.25.

### 2.8. Principal Component Analysis

Principal component analysis is a commonly used dimension reduction technique that aims at finding the linear transformation of the initial data space into the so-called ‘components’ space which best explains the data variance. More formally, let X be the data matrix of dimensions n×p where *n* is the number of observations (i.e., the number of TACs) and *p* the number of variables (i.e., the number of samples per TAC). The method aims at finding the new axis system $\left(e_{1},e_{2},\cdots ,e_{p}\right)$ in which the variance

(11) $$s_{e_{i}}^{2}=e_{i}^{⊤}S\;e_{i}$$

is maximized under constraints $e_{i}^{⊤}e_{j}=\delta_{i,j}$ and $s_{e_{1}}^{2}\geq s_{e_{2}}^{2}\geq \cdots \geq s_{e_{p}}^{2}$, with $\delta_{i,j}$ being the Kronecker delta and S the data covariance matrix given by:

(12) $$S=\frac{1}{n-1}\;X_{c}^{⊤}X_{c},$$

where $X_{c}$ is the centered data matrix, i.e., the data matrix from which variable means have been subtracted columnwise. It can be shown that such components are given by the eigenvectors of S ordered by decreasing eigenvalues, corresponding to the respective component variances. As the amount of explained data variance decreases with the component number, it is expected that a sufficiently high amount of data variance can be explained by the first m≪p components, which is the case for highly correlated initial variables.

Our real and synthetic TAC datasets were split into PCA model-building and evaluation sets on a patient basis in order to evaluate the inter-patient generalization performance of the PCA model built. The model-building and evaluation sets comprise TACs from 20 (patients 1 to 20 in Table A1) and 13 (patients 21 to 33 in Table A1) patients, respectively, totaling 3,974,466 and 2,446,068 TACs.

The impact of noise on the true signal reconstruction and on the definition of the first 6 principal components was first investigated using the synthetic TAC dataset. For each level of noise, the principal components were first determined on the noisy model-building TAC set (20 patients, 3,974,466 TACs). The component values were then computed for each noisy evaluation TAC (13 patients, 2,446,068 TACs) at the considered noise level. Estimated denoised TACs were finally reconstructed from the first 4 and 6 component values and compared to the corresponding true unnoisy TAC using the mean squared error. To assess the robustness of our approach in the presence of atypical kinetics, the same numerical experiment was conducted by considering tumor evaluation TACs only (i.e., the 39,780 TACs extracted from the delineated BTVs). The influence of the noise level on the component definition was also assessed by computing the cosine between each of the first 6 principal components determined on the noisy model-building dataset and the corresponding component obtained for a noise level of zero.

The first 6 principal components were then determined on the real model-building TAC set (20 patients, 3,974,466 TACs) and compared to these determined on the synthetic model-building dataset for a noise level of zero by computing their respective cosine. The component values were then computed for each TAC of the 13 evaluation patients and mapped spatially to their respective voxel location. The possible dependence between the minimum, maximum, and mean values of the resulting PC maps within the BTV versus the 1p/19q codeletion and IDH mutation status was investigated by means of Mann–Whitney U tests with a significance level set to 0.05. The PC maps were finally compared to their homologous 1TCM kinetic parameter maps, visually and by means of Spearman’s correlation analyses for the 1TCM parametric maps.

Due to the limited amount of memory available, the incremental PCA (IPCA) algorithm was used for all analyses instead of classical matrix decomposition since it allows a batch processing of the data. The implementation used is available in Python as part of the scikit-learn’s ‘decomposition’ module [35]. No noise normalization was performed prior to PCA in this work.

## 3. Results

### 3.1. Principal Components

#### 3.1.1. Synthetic Data

**Figure 3:**
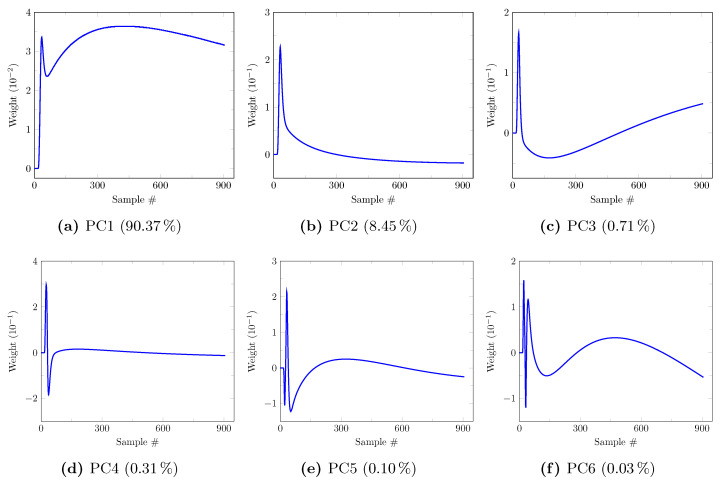
First 6 principal components (PCs) with their explained variance ratio determined on the synthetic model-building dataset (20 patients, 3,974,466 TACs) for a noise level of zero. Each component assigns a weight to each of the 906 TAC samples, starting from 10 s to 1820 s with step 2 s.

The first six principal components (PCs) determined on the synthetic model-building dataset (20 patients, 3,974,466 TACs) for a noise level of zero are depicted in Figure 3. Their corresponding explained variance ratios are, respectively, 90.37%, 8.45%, 0.71%, 0.31%, 0.10%, and 0.03% for PC1 to 6, totaling 99.97% of the explained variance. It should be noted that PC5 and PC6 have very low contribution to the explained variance. A clinical interpretation of the PCs can be proposed based on their respective shape. Aside from its early peak, PC1 (Figure 3a) assigns a relatively constant weight to the later samples and thus partly reflects the mean tracer uptake. PC2 (Figure 3b) overweights the early TAC samples, then assigns a rapidly decaying weight to the next samples with a slightly negative value for the last samples. This component thus has a high value for TACs with a high early activity as observed in blood after bolus injection of the tracer. PC3 (Figure 3c) assigns a negative weight to the first half of the samples and a positive weight to its second half, with a quasi-linear increase. It thus has a negative value for decreasing TACs, a positive value for increasing TACs and a value around zero for flat TACs. PC4 (Figure 3d) assigns a strongly positive weight to the very first samples, a strongly negative weight to the next few samples then a small quasi-constant weight to the last samples. PC4 thus assigns a negative value to TACs with delayed arterial peak and a positive value to TACs with non-delayed arterial peak. PC5 and 6 (Figure 3e,f) are less easily interpreted but together only explain 0.13% of the variance.

**Figure 4:**
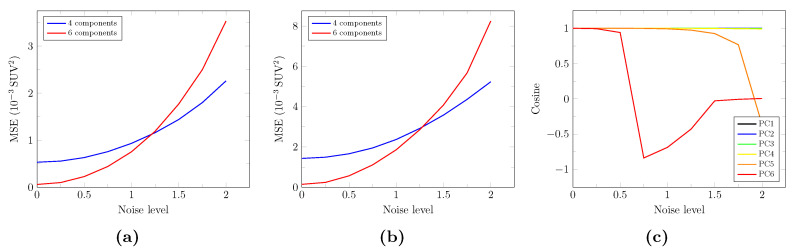
Influence of the noise level on the true signal reconstruction and on the definition of the first 6 principal components for the synthetic dataset. Mean squared error (MSE) between the true and estimated unnoisy TACs reconstructed from 4 (blue curves) and 6 (red curves) components for different noise levels, computed on the whole synthetic evaluation TAC set (**a**) and on the tumor synthetic evaluation TACs only (**b**). (**c**): Cosines between each principal component computed for noise levels ranging from 0 to 2.0 with step 0.25 and the corresponding component computed for a noise level of zero.

Figure 4 depicts the influence of the noise level on the true unnoisy signal reconstruction, assessed by the mean squared error computed on the whole synthetic evaluation TAC set (Figure 4a—2,446,068 TACs) and on the tumor synthetic evaluation TACs only (Figure 4b—39,780 TACs). Interestingly, signal reconstruction with 6 components is less accurate than that with 4 components for noise levels above 1.25 for both whole brain and tumor TACs. The twofold absolute squared errors observed for the tumor evaluation TACs (Figure 4b) compared to the whole evaluation TAC set (Figure 4a) are imputed to the higher global uptake of tumor voxels. Figure 4c depicts the impact of noise on the definition of the first 6 principal components, assessed by their cosine with the respective components determined for a noise level of zero (see Figure 3). The PC5 cosine progressively drops from a noise level of 1.0 whereas a rapid drop of the PC6 cosine is observed for noise levels above 0.5. PC1 to 4, on the other hand, remain stable even for high noise levels.

**Figure 5:**
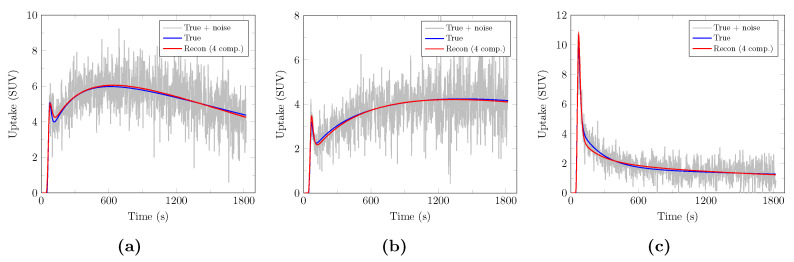
Examples of PCA-reconstructed synthetic evaluation TACs with 4 components for a realistic noise level of 1.5. Noisy synthetic TACs are depicted in light gray along with their corresponding true unnoisy TACs (blue) and PCA-denoised TACs with 4 components (red) for a decreasing (**a**), increasing (**b**), and arterial (**c**) TAC.

Examples of true, noisy, and PCA-denoised evaluation TACs with 4 components are depicted in Figure 5. Reconstruction with only 4 components is remarkably accurate while efficiently removing noise.

#### 3.1.2. Real Data

**Figure 6:**
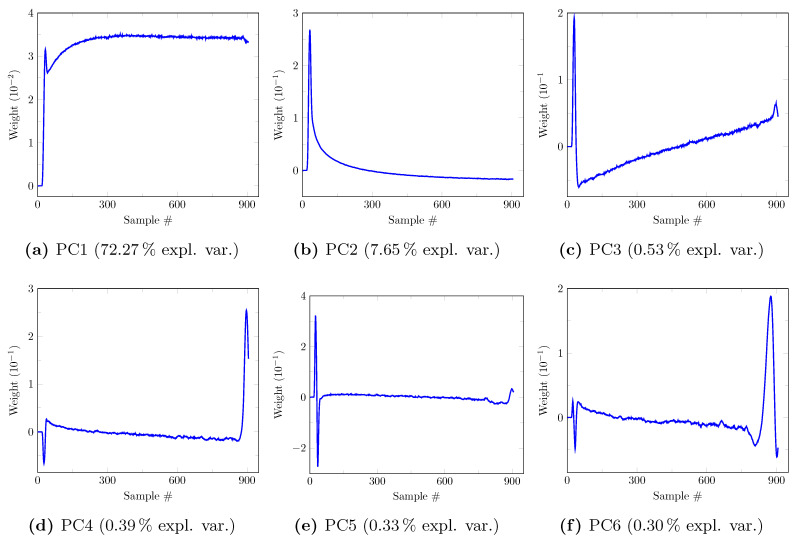
First 6 principal components (PCs) with their explained variance ratio determined on the real model-building dataset (20 patients, 3,974,466 TACs). Each component assigns a weight to each of the 906 TAC samples, starting from 10 s to 1820 s with step 2 s.

The first six principal components determined on the real model-building dataset are depicted in Figure 6. Their corresponding explained variance ratios are, respectively, 72.27%, 7.65%, 0.53%, 0.39%, 0.33%, and 0.30% for PC1 to 6, totaling 81.47% of the explained variance. Strong similarities are, respectively, observed between PC1–4 of the synthetic dataset (see Figure 3) and PC1–3 and 5 of the real dataset (see Figure 6), hence the same clinical interpretations can be made for the latter. These similarities are confirmed by their respective cosines of 0.9987, 0.9891, 0.9383, and 0.9111. The residual differences are suspected to originate from (i) the limited ability of the 1TCM used for data synthesis to fully capture the whole range of observed TAC dynamic behaviors and (ii) unmodeled additional sources of noise and artifacts related to data reconstruction and residual patient motion. It should be noted that PC4–6 of the real dataset have very similar explained variance ratios, hence their order was not considered informative as they could have been reversed for another similar dataset. Since PC4 and 6 of the real dataset are hardly interpretable and do not match any of the first six PCs of the synthetic dataset—as opposed to PC5—they will not be further considered from now on.

### 3.2. Parametric Maps

**Figure 7:**
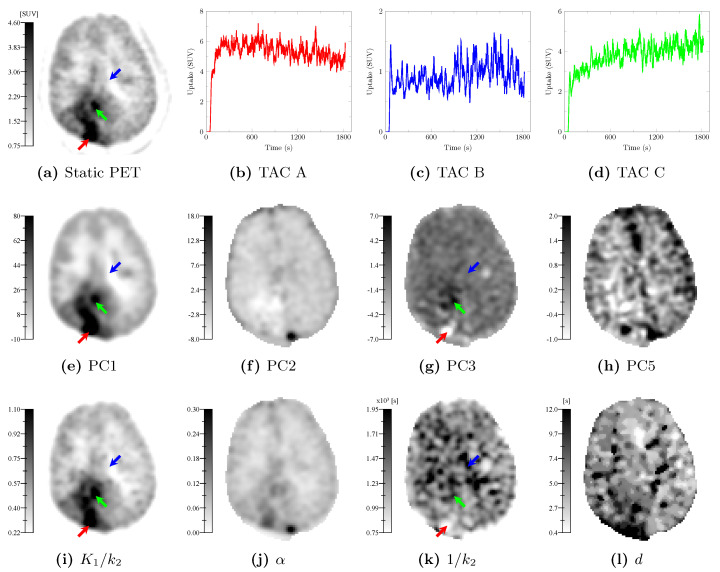
Parametric maps generated from the dynamic [^11^C]MET PET scan of a glioblastoma evaluation patient (patient 21 in Table A1). (**a**): Static PET image (20–27 min p.i.). (**b**–**d**): Smoothed TACs at voxels pointed by the red, blue, and green arrows, respectively. TAC A, B, and C, respectively, have a ‘decreasing’, ‘flat’, and ‘increasing’ behavior. (**e**–**h**): Mapped values of the principal components 1–3 and 5 determined on the real model-building dataset. (**i**–**l**): 1TCM kinetic parameter maps. All maps are displayed in inverted grayscale.

Hereafter, the PC definitions used are these determined on the real model-building dataset (see Figure 6). The parametric maps obtained by voxelwise mapping of the PC values and of the 1TCM kinetic parameters computed over the voxel TACs of a glioblastoma evaluation patient (patient 21 in Table A1) are depicted in Figure 7 in inverted grayscale. The static PET image (20–27 min p.i.) is depicted in Figure 7a. Curves A, B, and C (Figure 7b–d) correspond to the smoothed TACs at voxels pointed by the red, blue, and green arrows, respectively. The PC1 map (Figure 7e) exhibits strong similarities with the static PET image (Figure 7a) and the $K_{1}/k_{2}$ map (Figure 7i). The PC2 map (Figure 7f) exhibits strong similarities with the α map (Figure 7j). The PC3 (Figure 7g) and $1/k_{2}$ (Figure 7k) maps share visual similarities but structures are hardly distinguishable on the $1/k_{2}$ map as opposed to PC3. The PC5 (Figure 7h) and *d* maps (Figure 7l) exhibit fairly similar patterns with inverted contrast for PC5.

Furthermore, the PC3 map (Figure 7g) clearly highlights regions with characteristic dynamic behaviors, as illustrated by TACs A, B, and C. Voxels with fast increasing then decreasing TACs appear brighter on the PC3 map (red arrow), whereas progressively increasing TACs appear darker (green arrow). Voxels with relatively flat TACs appear in medium gray value on the PC3 map (blue arrow). In contrast, the homologous $1/k_{2}$ map fails to clearly highlight these behaviors, as illustrated by voxels pointed by the green and blue arrows, both appearing darker in Figure 7k.

**Figure 8:**
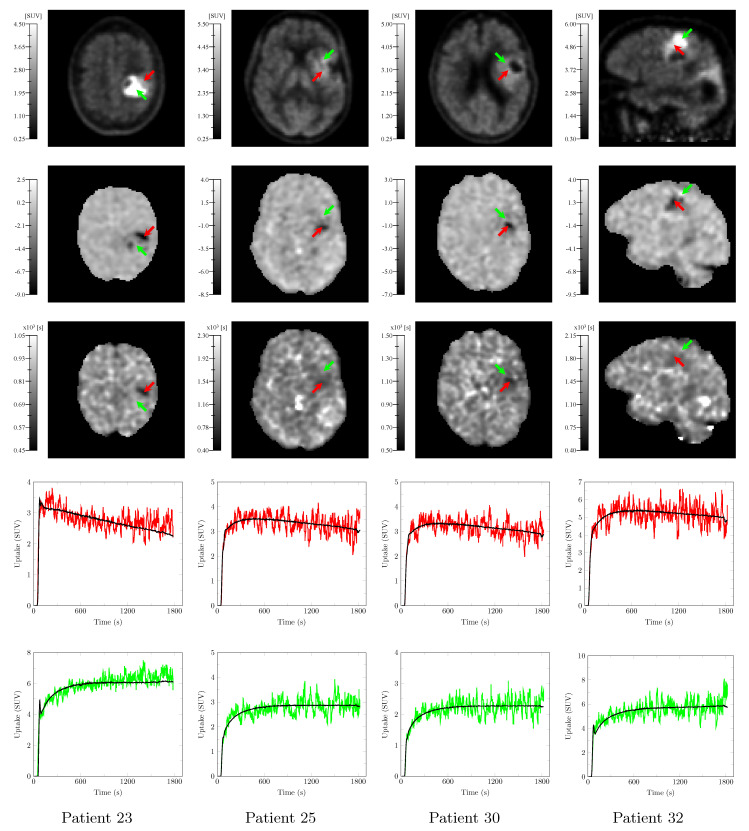
Static PET image (20–27 min p.i.—1st row) and PC3 (2nd row) and $1/k_{2}$ (3rd row) maps computed from the dynamic [^11^C]MET PET scans of four evaluation patients (patients 23, 25, 30, and 32 in Table A1) along with the smoothed TACs at voxels pointed by the red (4th row) and green (5th row) arrows, respectively. Red TACs have a ‘decreasing’ or ‘flat’ behavior whereas green TACs have a ‘flat’ or ‘increasing’ behavior. TAC reconstruction with 4 of the 5 principal components (PC1–3 and 5) is superimposed in black to each TAC.

Static PET images (20–27 min p.i.), PC3, and $1/k_{2}$ maps along with representative smoothed TACs at voxels pointed by the red, and green arrows are depicted in Figure 8 for 4 additional evaluation patients (patients 23, 25, 30, and 32 in Table A1). As opposed to the static PET image, the PC3 map allows to distinguish voxels with ‘decreasing’ or ‘flat’ TACs (red) from voxels with ‘flat’ or ‘increasing’ TACs (green). The homologous $1/k_{2}$ maps exhibits patterns similar to the PC3 maps within the BTV but with a substantially lower contrast. Similar behaviors were observed for the other eight evaluation patients. Interestingly, the substantial late uptake increase of the green TAC from patient 23 in Figure 8 is not totally captured by PC1–3 and 5 (see black curve) but PC4 and 6 are also required for a more accurate reconstruction.

**Figure A3:**
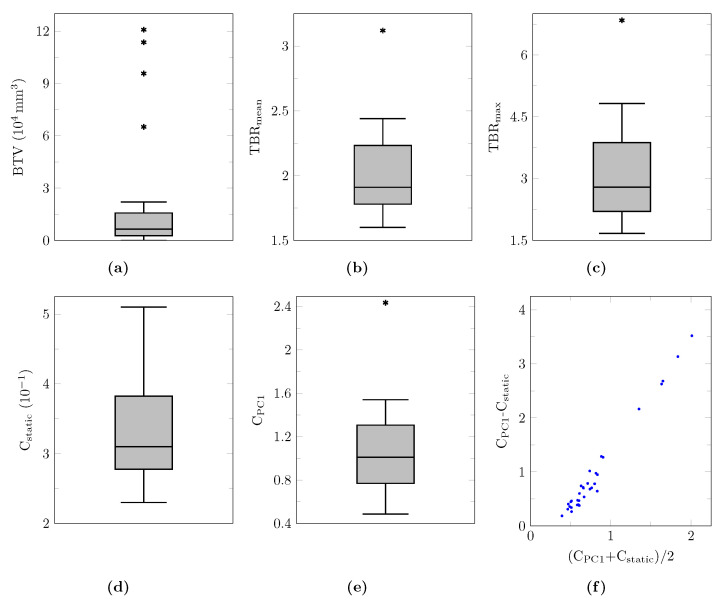
Distribution of the PET parameters in Table A3 for lesions with a non-zero BTV summarized by means of boxplots (horizontal line: median, box: interquartile range, whiskers: ±1.5 interquartile range, *: outliers) (**a**–**e**) and Bland-Altman plot of C_PC1_ versus C_static_ (**f**).

The biological tumor volume (BTV), mean and maximum tumor-to-background ratio (TBR) evaluated on the static PET image (20–27 min p.i.) as well as tumor contrast (see definition in Equation (A20)) values evaluated on the static PET image (C_static_) and on the PC1 map (C_PC1_) are reported for each lesion in Table A3. The corresponding distributions are summarized by means of boxplots in Figure A3a–e for lesions with a non-zero BTV. The Bland-Altman plot of C_PC1_ versus C_static_ is depicted in Figure A3f, illustrating the systematic higher tumor contrast of the PC1 maps compared to the static PET image.

**Figure A4:**
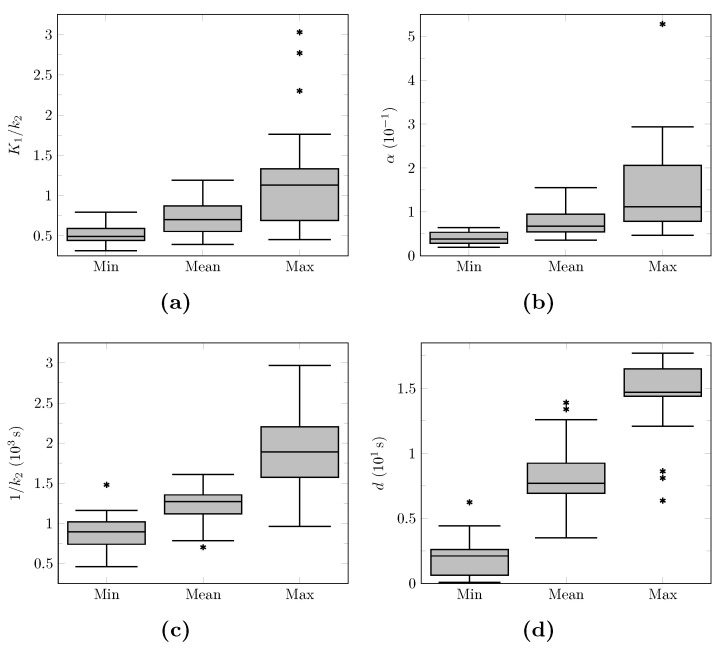
Distribution of the minimum, maximum, and mean kinetic parameter values within the BTV in Table A4 for lesions with a non-zero BTV summarized by means of boxplots (horizontal line: median, box: interquartile range, whiskers: ±1.5 interquartile range, *: outliers). (**a**): $K_{1}/k_{2}$. (**b**): α. (**c**): $1/k_{2}$. (**d**): *d*.

**Figure A5:**
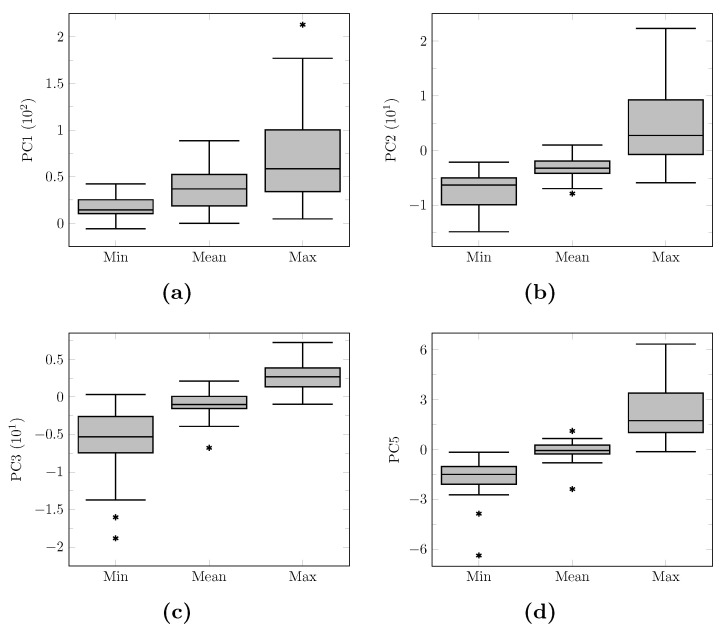
Distribution of the minimum, maximum, and mean PC values within the BTV in Table A5 for lesions with a non-zero BTV summarized by means of boxplots (horizontal line: median, box: interquartile range, whiskers: ±1.5 interquartile range, *: outliers). (**a**): PC1. (**b**): PC2. (**c**): PC3. (**d**): PC5.

**Figure A6:**
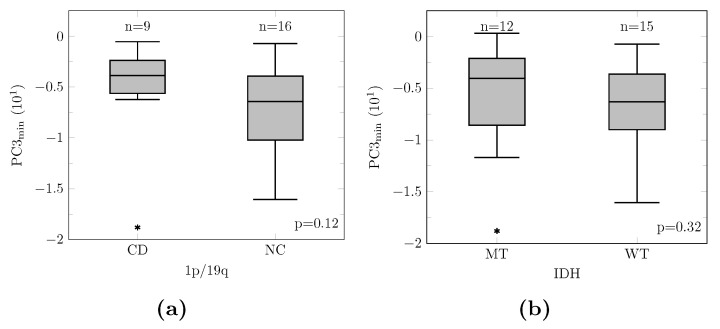
Boxplots of the minimum PC3 values within the BTV (horizontal line: median, box: interquartile range, whiskers: ±1.5 interquartile range, *: outliers) grouped by 1p/19q codeletion status (**a**) and IDH mutation status (**b**). The associated *p*-values were obtained by means of Mann–Whitney U tests. CD: codeleted, NC: non-codeleted, MT: mutant, WT: wildtype.

The minimum, maximum, and mean values of the 1TCM kinetic parameter maps and of the PC maps within the BTV are, respectively, reported for each lesion in Table A4 and Table A5. The corresponding distributions are summarized by means of boxplots in Figure A4 and Figure A5 for lesions with a non-zero BTV. None of the performed Mann–Whitney U tests investigating the dependence of the minimum, maximum, and mean PC values within the BTV were found statistically significant. Nevertheless, 1p/19q non-codeleted (*p* = 0.15) and IDH wildtype (*p* = 0.32) tumors tend to exhibit lower minimum PC3 values, as suggested by the grouped boxplots in Figure A6. It should be noted that removing the outlier (see asterisk mark in Figure A6a)—identified by a Grubb’s test (*p* < 0.05) performed after verification of the data normality by a Shapiro–Wilk test (*p* = 0.98)—leads to statistical significance for PC3_min_ versus the 1p/19q codeletion status (*p* = 0.04).

### 3.3. Parametric Map Correlations

The similarities between PC and 1TCM kinetic parameter maps observed are confirmed by the pairwise Spearman’s correlation coefficients computed voxelwise for each of the 13 evaluation patients taken individually and summarized in Table 1. Feature pairs PC2|α and PC5|*d* strongly correlate whereas PC1|$K_{1}/k_{2}$ and PC3|$1/k_{2}$ very strongly correlate. Moderate positive correlations are also found between PC1|α—imputed to the overweighting of the very first samples of PC1 (see Figure 6a)—and PC3|$K_{1}/k_{2}$. All associated *p*-values were <0.05—and <0.0001 for most of them due to the large number of analyzed voxels (∼200,000 voxels per patient scan)—except for pair PC5|$K_{1}/k_{2}$ of evaluation patient 33.

## 4. Discussion

We showed the ability of PCA to accurately capture characteristic dynamic behaviors from a broad spectrum of high dimensional [^11^C]MET PET TACs extracted from the whole brain region of 20 patients, most of whom have been treated multiple times. By means of realistic numerical simulations, we demonstrated the robustness of our approach to noise and validated the accuracy of the unnoisy signal reconstruction on a separate synthetic evaluation dataset not used for PCA model building, without the need for prior noise normalization. A possible explanation for this unexpected robustness to noise is that only TACs within the brain region were considered for this work—as opposed to the works of Pedersen and colleagues [20] and Šámal and colleagues [21]—hence background noise had no influence on the computed components.

Four components (PC1–3 and 5) among the six that best explain variations observed among the real whole brain TACs of 20 patients respectively matched the first four principal components derived from our synthetic model-building dataset. These four components have been found to be of clinical interest. PC1 provides a contrast similar to the routinely acquired static PET image (20–27 min p.i.) but has two advantages over the latter: (i) the PC1 map has a higher tumor contrast (see definition in Equation (A20)) than the static PET image, as shown in Figure A3f and (ii) PC1 does not depend on an arbitrary chosen imaging timing since each of the acquired samples starting from tracer injection contributes to some extent to the component value, making it more robust to inter-protocol variations. PC2 reflects the voxel vascular fraction but its small negative weighting of the late samples (see Figure 6b) makes interpretation less straightforward in tumor regions where high late uptake negatively influences the component value. PC3 provides an interesting dynamic contrast related to the shape of the tissue TAC, distinguishing voxel with fast increasing then decreasing, flat, or progressively increasing TACs. PC5 appears to be related to the relative blood delay and could reflect the effectiveness of micro-vascularization, which is known to be impaired in gliomas [36].

These four PCs strongly to very strongly correlate with the kinetic parameters of the widely used 1TCM PK model, which partly confirms our intuition concerning their clinical interpretation. On the other hand, the correlation between the kinetic parameters and PCs highlights the ability of a simplistic model such as the 1TCM to accurately capture the large variability observed among whole brain TACs of glioma patients. PCA has, however, three advantages over the 1TCM. First, it does not require a blood input function, avoiding invasive arterial sampling procedures or manual extraction of an IDIF potentially affected by PVE and tracer metabolites. Second, PCA model building is a parameter-free procedure, which makes it robust to inter-protocol variations. Third, the PCA parametric maps are much less computationally expensive to compute, with processing times of a few seconds for any new dynamic volume versus more than 3 h of fit for the 1TCM on a high-end computer. Alternatively, a basis function approach could be used for 1TCM parameters fitting to shorten processing times and improve stability [37] but at the expense of the precision on the kinetic parameter values and still longer processing times than the calculation of the component values. Furthermore, the PC3 map has been shown to outperform the homologous 1TCM $1/k_{2}$ map in differentiating voxels with ‘increasing’ and ‘decreasing’ TAC, which has been previously linked to the tumor aggressiveness as will be further discussed.

It should however be noted that the PCs must not been seen as a surrogate for the kinetic parameters since each PC may reflect a mixture of kinetic parameters (see off-diagonal correlations in Table 1) and of other factors not captured by the 1TCM. PCs instead capture the largest variability factors observed among TACs resulting from complex underlying biological processes, under an orthogonality constraint. An unequivocal link between one PC and one biologically relevant micro-parameter is not guaranteed either—nor is it the case for the kinetic parameters—which both capture complex amino acid transport processes dependent, among others, on the number of amino acid transporters along the endothelial and tumor cell membrane, the concentrations of all endogenous transporter shared substrates in every model compartment, and the relative sizes of the compartments and thus (over-) cellularity [38].

Our findings may nevertheless have important implications for clinical management of gliomas: PCA applied to dynamic [^11^C]MET PET data is likely to provide additional quantitative spatial information on glioma heterogeneity. In particular, the PC3 map provides a novel contrast complementary to the static PET image that can help distinguishing voxels with similar late uptake values but different uptake time courses, as illustrated in Figure 8. This component translates the ‘increasing’ and ‘decreasing’ behavior of TACs observed of Pöpperl and colleagues at the whole tumor level [8] into a quantitative voxelwise metric. This metric may partly reflect biologically relevant parameters such as local over-expression of LAT1—an important amino acid transporter over-expressed in gliomas at both the blood–brain barrier and tumor cell level [39,40] transporting both [^11^C]MET [39,41] and [^18^F]FET [41]. LAT1 being an obligatory exchanger, its over-expression may indeed be associated with an increase in both the influx and efflux of the tracer [41], leading to faster uptake dynamics that more closely follows the arterial input signal, hence characterized by a lower PC3 value. If this hypothesis turned out to be verified, application of the proposed methodology to dynamic [^18^F]FET data would also be of interest with an even more pronounced effect expected since [^18^F]FET is more specifically transported by LAT1 [42] and is not incorporated into proteins [15,41]. However, the possible relation between PC3 and the expression of LAT1 still needs to be verified, e.g., by means of targeted biopsies. Although not statistically significant, our preliminary analysis results are encouraging as they suggest that 1p/19q non-codeleted and IDH wildtype tumors may be characterized by lower minimum PC3 values (see Figure A6) and would thus exhibit more ‘decreasing’ TACs. These findings are in accordance with the previous work of Vetterman and colleagues on dynamic [^18^F]FET PET in gliomas, which reported a shorter time-to-peak in IDH wild type tumors [13]. However, relations between the patient outcome or glioma molecular features such as the IDH mutation or 1p/19q codeletion status and the distribution of the PC values within the BTV should be further investigated as part of a larger scale study with stricter inclusion criteria in terms of undergone treatments and subgroup balance, but it was out of the scope of this preliminary methodological work. To the best of our knowledge, this is the first work to promote the added value of dynamic PET acquisitions with [^11^C]MET in glioma patients to assess intra-tumor heterogeneity, as previously demonstrated for [^18^F]FET.

From a clinical point of view, our approach has the advantage of requiring only little modifications of the routine protocol, that is a longer acquisition time on the PET/CT tomograph but identical total duration of the procedure for the patient and no arterial sampling. Instead of injecting the patient outside the scanner and wait for a post injection delay before the acquisition, our preliminary results indeed support that additional information on the local tracer uptake dynamics can be obtained by injecting the patient inside the scanner right after the acquisition start without further modification of the protocol. The main drawback of the approach is related to the longer scanner occupancy, which would nevertheless have little impact if the procedure is reserved to the first patient of the day, selected accordingly. Besides, scanner occupancy will tend to be less problematic in the future with the advent of hybrid PET/MR scanners, which offer more time for PET acquisition as the MR acquisition typically constrains the total duration of the procedure. Once the acquisition is completed, the reconstruction and computation of the PC maps from a pre-built PCA model can be fully automatized as it does not require any manual processing and the results could be sent to the archiving system along with the static PET image. Although beyond the scope of this methodological work, potential clinical applications of the derived PC maps are multiple. The maps could first be introduced as an additional source of information for the definition of the resection margins or dose planning in radiotherapy—areas with an atypical dynamic behavior being potentially preferable to include in the clinical target volume. Additionally, the maps could be used for biopsy targeting in heterogeneous tumors such as gliomas with brighter or darker foci being possibly characterized by more aggressive histological features, reducing the risk of tumor under-grading. The analysis of staggered PC maps could also provide information on the treatment response over time. Finally, the PC maps may potentially provide additional information to distinguish glioma recurrence from radio-necrosis post radiotherapy.

The pharmacokinetic analyses conducted as part of this work were however prone to several limitations. Indeed, the decision was made for this study not to perform arterial sampling which requires a dedicated device and poses risks for the patient and the nursing staff. Instead, the blood input functions used for the kinetic analyses were extracted from the image within the petrous segment of the internal carotid arteries. As a consequence, the input functions used were affected by PVE and tracer metabolite activity, which could not be assessed individually for each patient. Population-based haematocrit and metabolite corrections as well as model-based spill-out coefficient estimation were thus proposed to counteract these effects. Moreover, spill-in correction of the IDIFs could not be trivially performed and is still a challenging open problem in PET imaging [43], which was out of the scope of this work. Nevertheless, it turned out in the course of this study that the introduction of the proposed input function corrections only affected the absolute values of the fitted kinetic parameters but that the contrast of the derived kinetic parametric maps was preserved. Since we were interested in this study in the relative quantification between voxels to highlight intra-tumor heterogeneity of the uptake dynamics, residual uncertainties in the corrected image-derived input functions were not suspected to significantly impact the conclusions of this work regarding the superiority of PCA to this extent. More complex pharmacokinetic models such as the two-tissue compartment model (2TCM) may also be considered to account for the tracer transport from the extra- to the intracellular space [44]. For longer scans, the synthesis of tracer metabolites in tissues—including proteins—would also need to be incorporated into the model as previously proposed for the assessment of the cerebral protein synthesis rate with L-[1-^11^C]leucine [45]. Nevertheless, as models grow in complexity, voxelwise fitting becomes even more challenging and prone to severe instabilities, as reported for the 2TCM [18]. In contrast, a model-free approach such as PCA has the advantage of being only concerned with extracting the highest variation factors of TACs, which could be as clinically relevant as model parameters if they turned out to be correlated with clinical or histological data.

For the sake of completeness, it should be noted that other dimension reduction techniques have been proposed in the literature for the analysis of dynamic PET data, such as factor analysis of dynamic structures (FADS) and non-negative matrix factorization (NMF) [46]. These are distinguished by their ability to isolate signal from noise, which strongly depends on the assumptions made on the noise distribution in PET images. Whereas the noise related to the event counting in PET imaging follows a Poisson distribution, the noise distribution in reconstructed PET images is less well characterized due to alterations related to the system hardware and reconstruction algorithm—including scatter and attenuation corrections [46]—hence remains an open problem. Comparison of these methods in the particular case of dynamic [^11^C]MET PET data of glioma patients would also be of interest as a future work.

## 5. Conclusions

We showed the ability of principal component analysis to extract meaningful parametric maps from noisy high dimensional dynamic [S-methyl-^11^C]methionine PET scans of glioma patients with little modification of the routine protocol. One of these maps was found to reflect at the voxel level the previously reported ‘increasing’ or ‘decreasing’ behavior of TACs within the tumor, which could potentially be linked to the aggressiveness heterogeneity within the tumor. Such maps could be of great interest for tumor characterization as well as for surgery and radiotherapy planning in addition to conventional static PET imaging. This early methodological work paves the way for many possible clinical studies.

## Figures and Tables

**Table 1:** Spearman’s correlation coefficients (median ± median absolute deviation) of all PC / kinetic parameter pairs computed voxelwise for each of the 13 evaluation patients taken individually.

	$K_{1}/k_{2}$	α	$1/k_{2}$	*d*
**PC1**	0.90±0.04	0.56±0.06	0.05±0.06	−0.02±0.05
**PC2**	−0.30±0.19	0.70±0.14	−0.34±0.05	−0.17±0.11
**PC3**	0.49±0.13	0.11±0.14	0.86±0.05	−0.26±0.11
**PC5**	−0.07±0.08	0.26±0.20	−0.31±0.08	−0.69±0.09

**Figure A1:**
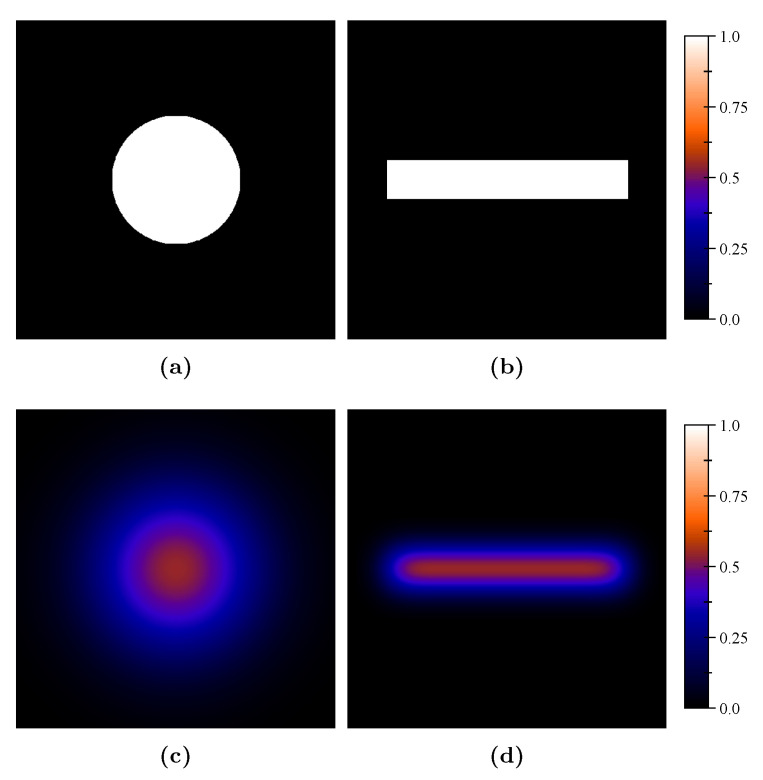
Results of the numerical convolution of the carotid model with the scanner PSF. Modeled petrous segment of the internal carotid artery filled with unit activity concentration in transverse (**a**) and axial (**b**) planes and corresponding simulated activity concentration after convolution with the scanner PSF modeled by an isotropic Gaussian kernel with standard deviation σ=1.9mm (**c**,**d**).

**Figure A2:**
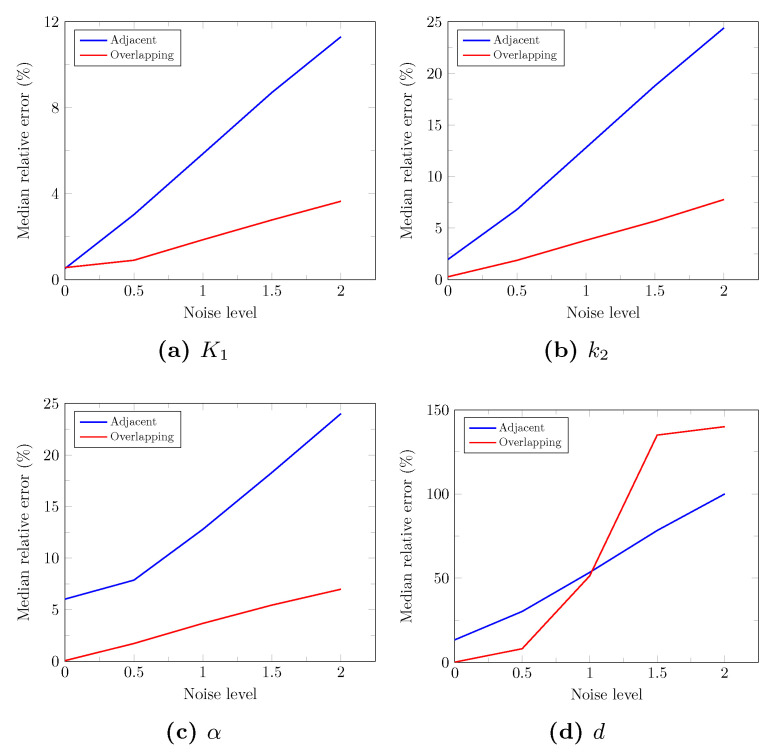
Median relative errors on the fitted 1TCM parameter values computed over the 10,000 simulated TACs for each level of noise and both framing strategies (blue curve: adjacent, red curve: overlapping).

## Data Availability

The data presented in this study are available on reasonable request from the corresponding author.

## Abbreviations

The following abbreviations are used in this manuscript:

[^11^C]MET	[S-methyl-^11^C]methionine
[^18^F]FET	O-(2-[^18^F]fluoroethyl)-L-tyrosine
1TCM	One-tissue compartment model
2TCM	Two-tissue compartment model
AIF	Arterial input function
BTV	Biological tumor volume
CT	Computed tomography
CTAC	Computed tomography-based attenuation correction
FADS	Factor analysis of dynamic structures
FLAIR	Fluid-attenuated inversion recovery
FWHM	Full-width at half-maximum
HGG	High-grade glioma
IDIF	Image-derived input function
IPCA	Incremental principal component analysis
LGG	Low-grade glioma
MRI	Magnetic resonance imaging
NMF	Non-negative matrix factorization
p.i.	Post-injection
PCA	Principal component analysis
PET	Positron emission tomography
PK	Pharmacokinetic
PVE	Partial volume effect
ROI	Region of interest
SNR	Signal-to-noise ratio
SUV	Standardized uptake value
TAC	Time-activity curve
TBR	Tumor-to-background ratio
TOF-OSEM	Time-of-flight ordered subset expectation maximization
TTP	Time-to-peak
VOI	Volume of interest
WHO	World Health Organization

## Appendix A. Supplementary Methods

### Appendix A.1. Patient Characteristics

Patient’s clinical data, 2016 WHO classification, and undergone treatments at imaging time are provided for each lesion in Table A1.

**Table A1:** Patient’s clinical data, location and 2016 WHO classification of each analyzed lesion, and undergone treatments at imaging time. F: female, M: male, N/A: not available, MT: mutant, WT: wildtype, CD: codeleted, NC: non-codeleted, TMZ: temozolomide, CCNU: lomustin, RT: conventional radiotherapy, GK: gamma knife. Multiple lesions were identified for patients 19, 24, and 32. Patients 1 to 20 and 21 to 33, respectively, belong to the PCA model-building and evaluation sets.

	Clinical			2016 WHO Classification				Treatments		
Patient	Age	Sex	Location	Histology	Grade	IDH1	1p/19q	Surgery	Chemotherapy	Radiotherapy
1	67	M	Right post-rolandic	Astrocytoma	III	MT	N/A	Yes	TMZ	RT
2	53	F	Right fronto-callosum	Glioblastoma	IV	MT	N/A	Yes	TMZ	RT
3	33	M	Left frontal	Oligodendroglioma	III	MT	CD	Yes	TMZ	RT
4	56	F	Right fronto-parietal	Astrocytoma	III	WT	NC	Yes	TMZ	RT, GK
5	47	F	Left frontal	Oligodendroglioma	III	MT	CD	Yes	TMZ	RT
6	23	M	Left parietal	Oligodendroglioma	III	MT	CD	Yes	TMZ	RT
7	48	M	Right pre-rolandic	Oligodendroglioma	II	MT	CD	Yes	TMZ	No
8	56	M	Right frontal	Glioblastoma	IV	MT	NC	Yes	No	No
9	63	M	Left temporal	Glioblastoma	IV	WT	NC	Yes	TMZ	RT
10	34	F	Thalamo-mesencephalic	Rosette-forming glioneural tumor	I	N/A	N/A	No	No	RT
11	36	F	Right frontal	N/A	N/A	N/A	N/A	No	No	No
12	52	F	Left frontal	Glioblastoma	IV	WT	NC	Yes	TMZ	RT, GK
13	51	F	Left parietal	Oligodendroglioma	III	N/A	CD	Yes	TMZ	RT
14	58	M	Right fronto-parietal	Glioblastoma	IV	WT	NC	Yes	TMZ	RT
15	56	F	Left frontal	Astrocytoma	III	MT	NC	Yes	TMZ	RT
16	50	M	Left frontal	Oligodendroglioma	III	MT	CD	Yes	TMZ	RT
17	61	M	Right fronto-temporal	Oligodendroglioma	II	MT	CD	Yes	TMZ	No
18	55	M	Right frontal	Astrocytoma	III	MT	NC	Yes	TMZ	GK
19	83	M	Brainstem Left fronto-insular	N/A N/A	N/A N/A	N/A N/A	N/A N/A	No No	TMZ	RT No
20	58	M	Right frontal	Glioblastoma	IV	WT	NC	Yes	TMZ	RT, GK
21	52	F	Right parieto-occipital	Glioblastoma	IV	WT	N/A	Yes	TMZ, CCNU	RT
22	50	M	Left temporal	Glioblastoma	IV	WT	NC	Yes	TMZ	RT
23	70	F	Left frontal	Glioblastoma	IV	WT	NC	No	No	No
24	35	F	Right frontal Left insular	Oligodendroglioma N/A	III N/A	MT N/A	CD N/A	Yes No	TMZ	RT No
25	55	F	Left temporal	Glioblastoma	IV	WT	NC	No	TMZ	RT
26	61	M	Right fronto-temporo-insular	Oligodendroglioma	II	MT	CD	Yes	TMZ	No
27	53	M	Left fronto-insular	Oligodendroglioma	III	MT	CD	Yes	TMZ	RT
28	53	M	Right parietal	Glioblastoma	IV	WT	NC	Yes	TMZ, CCNU	RT
29	60	M	Right frontal	Glioblastoma	IV	WT	NC	Yes	TMZ	RT
30	76	F	Left fronto-parietal	Glioblastoma	IV	WT	NC	Yes	TMZ	RT
31	42	M	Left frontal	Glioblastoma	IV	WT	NC	Yes	TMZ	RT
32	63	M	Right parieto-occipital Right frontal Left frontal	Glioblastoma Glioblastoma N/A	IV IV N/A	WT WT N/A	NC NC N/A	Yes Yes No	TMZ, CCNU	RT RT No
33	56	M	Right temporo-insular	Astrocytoma	III	MT	NC	Yes	TMZ	RT

### Appendix A.2. Spill-Out Estimation

The spill-out estimation for IDIF correction was performed both analytically and by means of computer simulations. For the sake of simplicity, the scanner resolution was considered spatially constant and isotropic, characterized by a full-width at half-maximum (FWHM) of 4.5 mm consistent with our scanner specification. The scanner PSF *g* was modeled by a 3D isotropic Gaussian with standard deviation $\sigma =\mathrm{FWHM}/\left(2\sqrt{2\ln(2)}\right)\approx 1.91\;\mathrm{mm}$. The petrous segment of the carotid artery from which the whole blood input function is extracted in the image was modeled by a cylinder with radius R=2.4mm [47] and height H=30mm, determined experimentally based on time-of-flight MR data. For the calculation of the spill-out coefficient, a homogeneous activity distribution f(x,y,z) was constructed for such a cylinder centered at the origin, given by:

(A1) $$f\left(x,y,z\right)=\left\{\begin{array}{cc} 1, & \mathrm{if}\;(x,y,z)\in \mathrm{cylinder}\\ 0, & \mathrm{otherwise}\; \end{array}\right..$$

The convolution *c* of *f* with the scanner PSF *g* at point (x,y,z) is then given by:

(A2) $$\begin{array}{cc} c(x,y,z) & =f(x,y,z)*g(x,y,z) \end{array}$$

(A3) $$\begin{array}{cc} \; & =\int_{t}\int_{u}\int_{v}f\left(t,u,v\right)\;g\left(x-t,y-u,z-v\right)\;dv\;du\;dt \end{array}$$

(A4) $$\begin{array}{cc} \; & =\frac{1}{{\left(2\pi \sigma^{2}\right)}^{\frac{3}{2}}}\;\int_{t}\int_{u}\int_{v}f\left(t,u,v\right)\;e^{-\frac{{\left(x-t\right)}^{2}+{\left(y-u\right)}^{2}+{\left(z-v\right)}^{2}}{2\sigma^{2}}}\;dv\;du\;dt. \end{array}$$

To solve Equation (A4), the problem was re-expressed in cylindrical coordinates using the following change of variables:

(A5) $$\begin{array}{cc} t & =r\;\cos\theta , \end{array}$$

(A6) $$\begin{array}{cc} u & =r\;\sin\theta , \end{array}$$

(A7) $$\begin{array}{cc} v & =q, \end{array}$$

leading to:

(A8) $$c\left(x,y,z\right)=\frac{1}{{\left(2\pi \sigma^{2}\right)}^{\frac{3}{2}}}\;\int_{0}^{R}\int_{-\pi }^{\pi }\int_{-\frac{H}{2}}^{\frac{H}{2}}r\;e^{-\left(\frac{{\left(x-r\cos\theta \right)}^{2}+{\left(y-r\sin\theta \right)}^{2}+{\left(z-q\right)}^{2}}{2\sigma^{2}}\right)}\;dq\;d\theta \;dr.$$

Since no generic analytical solution exists for Equation (A8) at every point (x,y,z), we computed the spill-out coefficient at the central point (0,0,0) of the cylinder where it is supposed to be maximum, which gives:

(A9) $$\begin{array}{cc} c(0,0,0) & =\frac{1}{{\left(2\pi \sigma^{2}\right)}^{\frac{3}{2}}}\;\int_{0}^{R}\int_{-\pi }^{\pi }\int_{-\frac{H}{2}}^{\frac{H}{2}}r\;e^{-\frac{r^{2}\cos^{2}\theta +r^{2}\sin^{2}\theta +q^{2}}{2\sigma^{2}}}\;dq\;d\theta \;dr, \end{array}$$

(A10) $$\begin{array}{cc} \; & =\frac{1}{{\left(2\pi \sigma^{2}\right)}^{\frac{3}{2}}}\;\int_{0}^{R}\int_{-\pi }^{\pi }\int_{-\frac{H}{2}}^{\frac{H}{2}}r\;e^{-\frac{r^{2}}{2\sigma^{2}}}\;e^{-\frac{q^{2}}{2\sigma^{2}}}\;dq\;d\theta \;dr, \end{array}$$

(A11) $$\begin{array}{cc} \; & =\frac{1}{{\left(2\pi \sigma^{2}\right)}^{\frac{3}{2}}}\;\frac{{\left(\pi \sigma^{2}\right)}^{\frac{1}{2}}}{2^{\frac{1}{2}}}\left(\mathrm{erf}\left(\frac{\frac{H}{2}}{\sqrt{2}\sigma }\right)-\mathrm{erf}\left(\frac{-\frac{H}{2}}{\sqrt{2}\sigma }\right)\right)\int_{0}^{R}\int_{-\pi }^{\pi }r\;e^{-\frac{r^{2}}{2\sigma^{2}}}\;d\theta \;dr, \end{array}$$

(A12) $$\begin{array}{cc} \; & =\frac{1}{{\left(2\pi \sigma^{2}\right)}^{\frac{3}{2}}}\;\frac{{\left(\pi \sigma^{2}\right)}^{\frac{1}{2}}}{2^{\frac{1}{2}}}\left(\mathrm{erf}\left(\frac{\frac{H}{2}}{\sqrt{2}\sigma }\right)-\mathrm{erf}\left(\frac{-\frac{H}{2}}{\sqrt{2}\sigma }\right)\right)\;2\pi \;\int_{0}^{R}r\;e^{-\frac{r^{2}}{2\sigma^{2}}}\;dr, \end{array}$$

(A13) $$\begin{array}{cc} \; & =\frac{1}{{\left(2\pi \sigma^{2}\right)}^{\frac{3}{2}}}\;\frac{{\left(\pi \sigma^{2}\right)}^{\frac{1}{2}}}{2^{\frac{1}{2}}}\left(\mathrm{erf}\left(\frac{\frac{H}{2}}{\sqrt{2}\sigma }\right)-\mathrm{erf}\left(\frac{-\frac{H}{2}}{\sqrt{2}\sigma }\right)\right)\;2\pi \;\sigma^{2}\left(1-e^{-\frac{R^{2}}{2\sigma^{2}}}\right), \end{array}$$

(A14) $$\begin{array}{cc} \; & =\frac{1}{2}\;\left(1-e^{-\frac{R^{2}}{2\sigma^{2}}}\right)\;\left(\mathrm{erf}\left(\frac{\frac{H}{2}}{\sqrt{2}\sigma }\right)-\mathrm{erf}\left(\frac{-\frac{H}{2}}{\sqrt{2}\sigma }\right)\right). \end{array}$$

Provided that an analytical expression can be found for Equation (A8), the average spill-out coefficient $c_{\mathrm{voxel}}$ for a voxel of size 2 mm × 2 mm × 2 mm at the center of the petrous segment of the internal carotid would have been obtained by:

(A15) $$c_{\mathrm{voxel}}=\frac{1}{8}\;\int_{-1}^{1}\int_{-1}^{1}\int_{-1}^{1}c\left(x,y,z\right)\;dz\;dy\;dx.$$

Alternatively, a numerical approximation of $c_{\mathrm{voxel}}$ was computed by convolution of a 3D image with voxel size 0.02 mm × 0.02 mm × 0.02 mm in the center of which a cylinder of radius *R* and height *H* was drawn with a 3D isotropic Gaussian kernel with standard deviation σ/0.02 pixels truncated at 4 σ. An approximated value of $c_{\mathrm{voxel}}$ was obtained by averaging the 100 × 100 × 100 central voxels in the convolved image.

### Appendix A.3. Numerical Simulations

Taking into account the carotid-to-voxel delay *d*, the analytical expression of the 1TCM tissue tracer activity $c_{t}\left(t\right)$ for t≥d and the plasma input function in Equation (9) is given by:

(A16) $$\begin{array}{cc} c_{t}\left(t\right) & =h_{1}\left(t\right)*c_{p}\left(t-d\right),\\ \; & =\frac{1}{1-h}\;\left[\frac{A_{1}\;K_{1}}{k_{2}-\lambda_{1}}\left(m\;{\left(t-d\right)}^{2}+\left(p+\frac{m}{k_{2}-\lambda_{1}}\right)\left(t-d\right)\right)e^{-\lambda_{1}\left(t-d\right)}\right.\\ \; & +\left.\sum_{i=1}^{3}\;\frac{{\tilde{A}}_{i}\;K_{1}}{k_{2}-\lambda_{i}}\;\left[\left(p-\frac{m}{k_{2}-\lambda_{i}}\right)\left(e^{-\lambda_{i}\left(t-d\right)}-e^{-k_{2}\left(t-d\right)}\right)+m\;\left(t-d\right)\;e^{-\lambda_{i}\left(t-d\right)}\right]\right.\\ \; & +\left.\frac{A_{1}\;K_{1}\;m}{{\left(k_{2}-\lambda_{1}\right)}^{3}}\;\left(e^{-\lambda_{1}\left(t-d\right)}-e^{-k_{2}\left(t-d\right)}\right)\right],\\ \tilde{A} & =[-A_{2}-A_{3}-\frac{A_{1}}{K_{1}-\lambda_{1}},A_{2},A_{3}]. \end{array}$$

The mean voxel activity concentration $c_{v}^{t_{s}\to t_{e}}$ for a frame starting at time $t_{s}$ and ending at time $t_{e}$ is then given by:

(A17) $$c_{v}^{t_{s}\to t_{e}}=\frac{1}{t_{e}-t_{s}}\;\left(\alpha \;\int_{t_{s}}^{t_{e}}c_{b}\left(t-d\right)\;dt+\left(1-\alpha \right)\;\int_{t_{s}}^{t_{e}}c_{t}\left(t\right)\;dt\right),$$

where $c_{b}\left(t\right)$ and $c_{t}\left(t\right)$ are, respectively, given by Equations (A18) and (A19):

(A18) $$\begin{array}{cc} \int_{t_{s}}^{t_{e}}c_{b}\left(t-d\right)\;dt & =\frac{A_{2}+A_{3}-A_{1}\;\left(t_{e}-d+\frac{1}{\lambda_{1}}\right)}{\lambda_{1}}\;e^{-\lambda_{1}\left(t_{e}-d\right)}\\ \; & -\frac{A_{2}+A_{3}-A_{1}\;\left(t_{s}-d+\frac{1}{\lambda_{1}}\right)}{\lambda_{1}}\;e^{-\lambda_{1}\left(t_{s}-d\right)}\\ \; & -\sum_{i=2}^{3}\;\frac{A_{i}}{\lambda_{i}}\;\left(e^{-\lambda_{i}\left(t_{e}-d\right)}-e^{-\lambda_{i}\left(t_{s}-d\right)}\right), \end{array}$$

(A19) $$\begin{array}{c} \begin{array}{cc} \int_{t_{s}}^{t_{e}}c_{t}\left(t\right)\;dt & =\frac{1}{1-h}\;\left[\frac{A_{1}\;K_{1}}{k_{2}-\lambda_{1}}\left(\frac{m\;\lambda_{1}^{2}\;{\left(t_{s}-d\right)}^{2}+\lambda_{1}\;\gamma \;\left(t_{s}-d\right)+\gamma }{\lambda_{1}^{3}}\;e^{-\lambda_{1}\left(t_{s}-d\right)}\right.\right.\\ \; & -\left.\left.\frac{m\;\lambda_{1}^{2}\;{\left(t_{e}-d\right)}^{2}+\lambda_{1}\;\gamma \;\left(t_{e}-d\right)+\gamma }{\lambda_{1}^{3}}\;e^{-\lambda_{1}\left(t_{e}-d\right)}\right)\right.\\ \; & +\left.\sum_{i=1}^{3}\;\frac{{\tilde{A}}_{i}\;K_{1}}{k_{2}-\lambda_{i}}\left[\frac{p-\frac{m}{k_{2}-\lambda_{i}}}{k_{2}}\left(e^{-k_{2}\left(t_{e}-d\right)}-e^{-k_{2}\left(t_{s}-d\right)}\right)\right.\right.\\ \; & +\left.\left.\frac{m\;\lambda_{i}\;\left(t_{s}-d\right)+\left(p-\frac{m}{k_{2}-\lambda_{i}}\right)\lambda_{i}+m}{\lambda_{i}^{2}}\;e^{-\lambda_{i}\left(t_{s}-d\right)}\right.\right.\\ \; & -\left.\left.\frac{m\;\lambda_{i}\;\left(t_{e}-d\right)+\left(p-\frac{m}{k_{2}-\lambda_{i}}\right)\lambda_{i}+m}{\lambda_{i}^{2}}\;e^{-\lambda_{i}\left(t_{e}-d\right)}\right]\right.\\ \; & +\left.\frac{A_{1}\;K_{1}\;m}{{\left(k_{2}-\lambda_{1}\right)}^{3}}\;\left(\frac{e^{-k_{2}\left(t_{e}-d\right)}-e^{-k_{2}\left(t_{s}-d\right)}}{k_{2}}-\frac{e^{-\lambda_{1}\left(t_{e}-d\right)}-e^{-\lambda_{1}\left(t_{s}-d\right)}}{\lambda_{1}}\right)\right], \end{array}\\ \gamma =\left(p+\frac{m}{k_{2}-\lambda_{1}}\right)\;\lambda_{1}+2\;m. \end{array}$$

### Appendix A.4. Overlapping Frames

To quantify the added value of overlapping frames over distinct adjacent frames for the estimation of the 1TCM kinetic parameters, the following numerical experiments were conducted. The Feng’s input function parameters in Equation (8) were first least-squares fitted for each of the 33 patients’ IDIF using the SciPy’s ‘optimize’ and ‘signal’ modules [32] in Python. Frame-averaged blood input functions were then computed for each of the 33 fitted Feng’s parameter values using Equation (A18) and for two distinct framing strategies: (i) 906 overlapping frames of length 20 s with a shift of 2 s (the framing used in this work) and (ii) 92 non-overlapping adjacent frames of length 20 s. For the same two framing strategies, frame-averaged voxel TACs were generated using Equations (A17)–(A19) for 10,000 combinations of kinetic parameter values randomly chosen among the whole set of kinetic parameter values previously fitted on the real patients TACs (33 patients, 6,420,534 curves) with the corresponding Feng’s parameter values. For each noise level in range 0.0 to 2.0 with step 0.5 and for both framing strategies, synthetic noise was added to the frame-averaged blood input functions and to each of the 10,000 frame-averaged voxel TACs using Equation (10). Kinetic parameter values were finally estimated for each pair of noisy frame-averaged blood input function and voxel TAC using our least-squares transfer function fitting routine in Python and the relative errors between ground truth and estimated kinetic parameter values were computed. To assess the robustness of our approach in the presence of atypical kinetics, the same numerical experiment was conducted by randomly choosing the 10,000 combinations of kinetic parameter values among these extracted from the BTVs only.

## Appendix B. Supplementary Results

### Appendix B.1. Spill-Out Estimation

The evaluation of Equation (A14) for respective values of *R* and *H* of 2.4 mm and 30 mm gave a maximum spill-out coefficient c(0,0,0)≈0.55. Numerical convolution results are depicted in Figure A1. The spill-out coefficient value of the central 0.02 mm × 0.02 mm × 0.02 mm simulation grid voxel was of 0.55, in accordance with the analytical value. The average spill-out coefficient value within the 100 × 100 × 100 central voxels of the simulation grid—corresponding to the central PET image voxel—was of 0.51. This value was used for IDIF correction prior to the PK analyses.

### Appendix B.2. Overlapping Frames

The median relative errors on the fitted 1TCM parameter values computed over the 10,000 simulated whole brain TACs are depicted in Figure A2 for each level of noise and both framing strategies. Except for the delay parameter *d*, overlapping frames systematically provide lower median relative errors. Furthermore, the reduction of the fitting error for overlapping compared to adjacent frames increases with the level of noise. The decrease in median error using overlapping frames for a realistic noise level of 1.5 was of 5.9%, 13.1%, and 12.9% for $K_{1}$, $k_{2}$, and α, respectively. The associated *p*-values returned by the Wilcoxon’s signed-rank test were not considered reliable due to the large number of samples (n=10,000), hence the Wilcoxon’s effect sizes are reported instead in Table A2. Similar curves were obtained when considering kinetics extracted from the BTVs only, with an overall lower median error for both strategies and all noise levels. The corresponding absolute decrease in the median error was of 2.7%, 7.3%, and 10.5% for $K_{1}$, $k_{2}$, and α at a noise level of 1.5, which confirms the robustness of our approach in the presence of atypical kinetics.

**Table A2:** Wilcoxon’s effect sizes of the relative error difference between the overlapping and adjacent framing strategies for each of the 1TCM kinetic parameters and each noise level. Negative values correspond to an increase in the absolute error for the overlapping framing with regard to the adjacent framing.

	Noise Level				
	0.0	0.5	1.0	1.5	2.0
$K_{1}$	0.12	0.72	0.70	0.70	0.70
α	0.87	0.77	0.72	0.72	0.72
$k_{2}$	0.87	0.74	0.71	0.71	0.70
d	0.87	0.70	−0.10	−0.67	−0.59

### Appendix B.3. PET Data Analysis

Table A3 reports for each lesion the biological tumor volume (BTV), the mean and maximum tumor-to-background ratio (TBR) evaluated on the static PET image (20–27 min p.i.) as well as the tumor contrast evaluated on the static PET image (C_static_) and on the PC1 (C_PC1_) map, given by:

(A20) $$C=\frac{|{\mathrm{tumor}}_{\mathrm{mean}}-{\mathrm{contralateral}}_{\mathrm{mean}}|}{|{\mathrm{tumor}}_{\mathrm{mean}}+{\mathrm{contralateral}}_{\mathrm{mean}}|},$$

where ${\mathrm{tumor}}_{\mathrm{mean}}$ and ${\mathrm{contralateral}}_{\mathrm{mean}}$ are, respectively, the mean value within the BTV and the contralateral spherical ROI (see Section 2.5). The corresponding distributions are summarized by means of boxplots in Figure A3a–e for lesions with a non-zero BTV. The Bland-Altman plot of C_PC1_ versus C_static_ is depicted in Figure A3f, illustrating the systematic higher tumor contrast of the PC1 maps compared to the static PET image.

The minimum, maximum, and mean values of the 1TCM kinetic parameters and of the PC values within the BTV are, respectively, reported for each lesion in Table A4 and Table A5. The corresponding distributions are summarized by means of boxplots in Figure A4 and Figure A5 for lesions with a non-zero BTV. Figure A6 depicts boxplots of the minimum PC3 values within the BTV grouped by 1p/19q codeletion status (Figure A6a) and IDH mutation status (Figure A6b).

**Table A3:** Results of the PET data analysis. BTV: biological tumor volume, TBR_max/mean_: maximum/mean tumor-to-background ratio computed on the static PET image (20–27 min p.i.), C_static/PC1_: tumor contrast computed on the static PET image/PC1 map.

Patient	BTV [mm^3^]	TBR_max_	TBR_mean_	C_static_	C_PC1_
1	0	-	-	-	-
2	32	1.67	1.60	0.23	1.25
3	664	2.46	1.90	0.31	2.99
4	2872	2.74	1.95	0.32	1.11
5	40	1.69	1.62	0.24	1.53
6	24	1.72	1.68	0.25	3.77
7	2920	2.21	1.79	0.28	0.74
8	120,808	4.80	2.35	0.40	0.79
9	1688	2.54	1.85	0.30	0.49
10	6992	2.46	1.90	0.31	0.91
11	928	1.99	1.73	0.27	3.40
12	95,744	4.82	2.22	0.38	0.64
13	0	-	-	-	-
14	2224	2.12	1.71	0.26	1.00
15	0	-	-	-	-
16	2880	2.17	1.74	0.27	1.54
17	4480	3.59	2.31	0.40	0.94
18	0	-	-	-	-
19	7080 9312	2.44 4.01	1.86 2.41	0.30 0.41	1.01 1.19
20	14,800	3.26	1.94	0.32	2.95
21	113,648	4.06	2.01	0.34	0.68
22	7000	2.79	1.97	0.33	1.30
23	21,976	6.84	3.12	0.51	1.15
24	65,104 648	3.77 2.03	2.09 1.75	0.35 0.27	0.83 0.67
25	8728	2.84	1.90	0.31	0.67
26	4896	3.83	2.37	0.41	1.11
27	18,440	3.50	2.17	0.37	0.84
28	6528	4.42	2.35	0.40	1.08
29	3240	2.18	1.75	0.27	2.43
30	4520	2.26	1.80	0.28	0.73
31	7288	3.08	1.91	0.31	1.01
32	10,880 21,416 5248	2.56 4.06 3.70	1.89 2.27 2.44	0.31 0.39 0.42	0.62 0.78 0.80
33	18,680	4.00	2.11	0.36	1.31

**Table A4:** Minimum, maximum, and mean 1TCM kinetic parameter values computed for each lesion within the BTV.

	$K_{1}/k_{2}$			α			$1/k_{2}$ [10^3^ s]			*d* [s]		
Patient	Min	Max	Mean	Min	Max	Mean	Min	Max	Mean	Min	Max	Mean
1	-	-	-	-	-	-	-	-	-	-	-	-
2	0.62	0.67	0.64	0.04	0.05	0.05	1.48	1.64	1.56	2.45	6.37	3.52
3	0.31	0.48	0.39	0.04	0.09	0.05	0.58	1.12	0.86	0.34	14.40	8.89
4	0.57	0.94	0.70	0.04	0.07	0.05	1.01	1.97	1.31	1.30	14.67	6.22
5	0.61	0.74	0.65	0.06	0.08	0.07	1.10	1.53	1.20	3.06	8.63	4.76
6	0.53	1.62	0.87	0.06	0.53	0.12	0.55	2.84	1.27	0.09	17.69	7.53
7	0.46	0.71	0.55	0.02	0.07	0.04	1.04	2.05	1.40	0.70	16.87	8.11
8	0.65	2.30	1.12	0.06	0.22	0.12	0.77	2.17	1.16	0.69	15.94	10.60
9	0.79	1.20	0.95	0.06	0.12	0.08	1.16	1.79	1.45	2.69	14.56	10.74
10	0.54	0.89	0.70	0.06	0.28	0.09	0.76	1.29	0.96	4.09	14.60	8.97
11	0.47	0.58	0.52	0.02	0.09	0.04	0.82	1.71	1.17	2.54	14.81	6.45
12	0.68	2.77	1.17	0.05	0.29	0.11	0.66	1.72	1.14	0.57	16.83	9.12
13	-	-	-	-	-	-	-	-	-	-	-	-
14	0.41	0.70	0.50	0.03	0.08	0.05	1.16	2.97	1.60	1.55	14.35	7.39
15	-	-	-	-	-	-	-	-	-	-	-	-
16	0.46	0.66	0.55	0.04	0.08	0.06	0.83	1.92	1.13	2.27	17.53	9.00
17	0.45	1.18	0.71	0.02	0.13	0.06	0.89	1.90	1.23	0.67	14.60	7.23
18	-	-	-	-	-	-	-	-	-	-	-	-
19	0.49 0.51	0.83 1.15	0.63 0.78	0.03 0.03	0.15 0.09	0.06 0.06	0.98 0.96	1.91 2.66	1.27 1.30	2.58 2.36	14.59 12.14	7.93 6.99
20	0.49	1.13	0.71	0.03	0.11	0.06	0.81	2.47	1.61	1.16	14.77	7.44
21	0.43	0.45	0.44	0.04	0.05	0.05	1.04	1.14	1.08	6.25	8.11	6.92
22	0.72	1.45	1.06	0.06	0.18	0.12	0.80	1.94	1.35	0.50	15.16	8.68
23	0.51	3.03	1.19	0.06	0.28	0.16	0.46	0.96	0.79	2.20	12.19	6.74
24	0.42 0.49	1.22 0.61	0.63 0.55	0.63 0.04	0.26 0.08	0.12 0.06	0.47 0.95	1.21 1.16	0.70 1.04	0.64 2.31	16.84 16.42	9.64 7.70
25	0.68	1.29	0.88	0.05	0.11	0.08	0.88	1.81	1.29	4.08	15.09	11.71
26	0.40	1.13	0.69	0.02	0.13	0.05	0.98	1.89	1.32	0.10	14.28	5.78
27	0.47	1.21	0.73	0.02	0.07	0.04	1.08	2.62	1.51	0.60	14.61	6.93
28	0.60	1.55	0.95	0.04	0.23	0.09	0.80	2.35	1.32	2.02	14.43	7.62
29	0.41	0.55	0.48	0.02	0.05	0.04	0.94	1.78	1.21	0.57	14.71	6.40
30	0.44	0.63	0.53	0.05	0.09	0.07	0.65	1.22	0.88	2.38	14.44	7.64
31	0.44	0.80	0.59	0.04	0.14	0.08	0.67	2.16	1.07	2.12	15.77	13.43
32	0.59 0.53 0.55	1.08 1.76 1.46	0.78 0.86 0.90	0.04 0.03 0.05	0.10 0.20 0.16	0.07 0.10 0.11	1.08 0.97 0.93	1.84 2.31 1.59	1.37 1.30 1.20	6.25 4.43 4.10	17.75 17.38 17.06	13.94 12.60 11.45
33	0.40	1.17	0.71	0.04	0.22	0.08	0.64	2.80	1.50	0.56	15.01	8.11

**Table A5:** Minimum, maximum, and mean PC values computed for each lesion within the BTV.

	PC1			PC2			PC3			PC5		
Patient	Min	Max	Mean	Min	Max	Mean	Min	Max	Mean	Min	Max	Mean
1	-	-	-	-	-	-	-	-	-	-	-	-
2	14.28	16.96	15.71	−5.36	−3.91	−4.69	0.33	1.39	0.81	−0.32	0.16	−0.11
3	4.27	28.78	16.24	−5.27	1.37	−3.17	−3.88	1.23	−1.00	−1.44	1.25	0.04
4	14.33	43.25	27.29	−7.01	−1.70	−4.39	−1.27	3.13	1.10	−1.97	1.24	−0.25
5	14.11	16.66	15.33	−4.75	−2.13	−3.04	−0.55	1.16	−0.07	−0.59	0.79	0.37
6	31.29	158.52	60.37	−9.53	40.13	−3.42	−13.74	7.23	0.79	−2.05	6.34	0.42
7	20.15	47.51	35.25	−6.44	2.69	−3.22	−4.21	1.66	−1.02	−1.67	1.58	0.24
8	28.37	170.40	71.41	−10.45	11.80	−3.25	−11.69	5.26	−1.16	−3.85	3.76	−0.72
9	41.57	83.09	58.68	−11.16	−5.85	−7.83	−1.02	4.11	2.11	−2.29	1.08	−0.48
10	22.72	55.64	36.90	−6.26	17.13	−2.05	−4.63	2.04	−1.09	−0.83	1.62	0.24
11	0.52	9.10	5.84	−4.09	2.80	−1.58	−2.46	3.27	0.30	−2.12	0.70	−0.79
12	42.19	212.93	88.56	−14.81	9.22	−4.26	−16.05	5.26	−2.21	−2.72	3.54	0.34
13	-	-	-	-	-	-	-	-	-	-	-	-
14	14.28	16.96	15.71	−5.36	−3.91	−4.69	0.33	1.39	0.81	−0.32	0.16	−0.11
15	-	-	-	-	-	-	-	-	-	-	-	-
16	14.28	16.96	15.71	−5.36	−3.91	−4.69	0.33	1.39	0.81	−0.32	0.16	−0.11
17	16.43	99.84	48.61	−10.55	8.43	−3.36	−5.43	5.80	−0.87	−2.25	1.92	−0.52
18	-	-	-	-	-	-	-	-	-	-	-	-
19	16.15 12.91	45.37 81.32	29.38 43.66	−4.35 −5.98	10.82 2.70	−1.09 −1.86	−3.89 −5.30	4.96 3.29	−1.37 −1.32	−1.55 −1.55	3.26 2.08	−0.30 −0.10
20	2.93	47.21	17.24	−5.62	3.56	−2.18	−5.51	2.85	0.25	−1.74	1.98	−0.22
21	4.87	5.68	5.31	−2.11	−1.67	−1.90	−1.54	−0.98	−1.34	−0.15	0.17	0.05
22	10.63	43.98	27.54	−4.97	5.63	−1.48	−5.64	3.19	−0.60	−1.25	3.54	0.08
23	11.16	176.62	64.48	−12.61	5.95	−4.10	−9.51	−0.18	−3.18	−6.36	−0.13	−2.37
24	24.51 23.93	126.14 35.83	53.75 29.72	−6.08 −7.02	13.17 −2.86	1.03 −5.03	−18.79 −1.16	1.11 1.05	−6.77 −0.03	−1.02 −0.72	4.55 0.62	1.12 −0.23
25	29.09	84.23	51.18	−6.44	0.59	−3.91	−6.31	2.70	−1.23	−1.49	1.58	−0.06
26	10.54	78.94	38.84	−9.88	9.33	−3.91	−2.66	4.21	1.12	−2.06	5.20	−0.05
27	22.11	101.12	51.98	−9.91	0.55	−4.96	−6.23	3.31	−1.43	−1.45	1.94	0.14
28	12.85	99.81	46.08	−4.82	22.30	0.41	−7.44	1.85	−2.99	−1.38	2.59	0.38
29	7.03	25.60	16.39	−4.05	0.21	−2.06	−4.56	0.76	−2.08	−0.97	1.51	0.19
30	25.53	57.29	39.09	−5.05	0.10	−2.52	−6.53	1.57	−1.99	−0.36	1.93	0.56
31	12.26	58.58	33.05	−4.99	6.12	−0.62	−12.30	2.52	−3.92	−1.01	4.51	0.64
32	29.90 25.09 26.66	75.90 137.01 119.75	51.22 63.97 71.06	−10.82 −10.30 −8.28	−2.89 2.77 3.39	−6.93 −4.88 −3.50	−3.31 −7.45 −5.64	2.67 3.76 1.21	0.02 −1.21 −1.90	−1.77 −2.20 −1.12	0.63 1.74 3.34	−0.55 12.60 0.67
33	9.00	75.38	30.90	−6.02	16.17	−1.39	−10.91	4.30	0.00	−2.54	4.81	−0.08

## Appendix C. Supplementary Discussion

We showed that the reconstruction of a large number of overlapping frames compared to a reduced number of adjacent frames of the same length benefits the estimation of the 1TCM kinetic parameters increasingly with the noise level, except for the blood delay *d*. Possible explanations for this gain are that: (i) overlapping frames provide a better sampling of the arterial peak for both blood and tissue TACs and (ii) an increased number of correlated data points helps the fitting algorithm to overcome noise. The latter remark does however not appear to hold for the estimation of the blood delay *d*, which consists of an arterial peak alignment process that could be negatively impacted by an increased number of early noisy TAC samples. Nevertheless, the blood delay *d* is rarely considered as a variable of interest in pharmacokinetic studies but is rather used to provide more flexibility to the fitting algorithm. The overlapping framing approach proposed has however two major drawbacks, that are (i) a longer reconstruction time and (ii) a larger memory usage for storage and processing. It should finally be noted that the overlapping framing used in this work may not be optimal for kinetic parameter values estimation. Determining the best dynamic PET framing strategy for pharmacokinetic studies would be of interest but is out of the scope of this paper.

We proposed to average the TACs of the 10 brightest voxels within the petrous segment of both internal carotid arteries on an early PET frame for blood input function extraction (see Section 2.3) since they were expected to be the least affected by PVE. It turned out in practice that these voxels are indeed almost systematically located along the central line of the segment, which is consistent with our simulations since the central line has the largest theoretical spill-out coefficient value. Whereas this method has the great advantage of not requiring an invasive arterial sampling, we showed hereabove that the activity within the central voxels is systematically underestimated by a factor near 2 due to partial volume effects resulting from the limited resolution of the scanner and the restricted diameter of the internal carotid arteries. We proposed to address this issue by scaling the extracted blood input functions by a factor 1/0.51≈1.96. This correction remains however limited since the geometry of the carotid arteries varies among patients [47]. Additionally, investigating the effects of these variations on the kinetic parameters estimation would also be of interest. Deriving a spill-in coefficient, in contrast, is less trivial since all TACs within the reconstructed volume are expected to impact the input function.

## References

[1] Lilja A., Bergström K., Hartvig P., Spännare B., Halldin C., Lundqvist H., Långstrom B. (1985). Dynamic Study of Supratentorial Gliomas with L-Methyl-11C-Methionine and Positron Emission Tomography. AJNR Am. J. Neuroradiol..

[2] De Witte O., Goldberg I., Wikler D., Rorive S., Damhaut P., Monclus M., Salmon I., Brotchi J., Goldman S. (2001). Positron Emission Tomography with Injection of Methionine as a Prognostic Factor in Glioma. J. Neurosurg..

[3] Sadeghi N., Salmon I., Decaestecker C., Levivier M., Metens T., Wikler D., Denolin V., Rorive S., Massager N., Baleriaux D. (2007). Stereotactic Comparison among Cerebral Blood Volume, Methionine Uptake, and Histopathology in Brain Glioma. AJNR Am. J. Neuroradiol..

[4] Goldman S., Levivier M., Brucher J.M., Wikler D., Damhaut P., Dethy S., Brotchi J., Hildebrand J. (1997). Regional Methionine and Glucose Uptake in High-Grade Gliomas: A Comparative Study on PET-Guided Stereotactic Biopsy. J. Nucl. Med..

[5] Pirotte B., Goldman S., David P., Wikler D., Damhaut P., Vandesteene A., Salmon I., Brotchi J., Levivier M. (1997). Stereotactic Brain Biopsy Guided by Positron Emission Tomography (PET) with [F-18]Fluorodeoxyglucose and [C-11]Methionine. Acta Neurochir. Suppl..

[6] Pirotte B., Goldman S., Massager N., David P., Wikler D., Vandesteene A., Salmon I., Brotchi J., Levivier M. (2004). Comparison of 18F-FDG and 11C-Methionine for PET-Guided Stereotactic Brain Biopsy of Gliomas. J. Nucl. Med..

[7] Pirotte B., Levivier M., Goldman S., Massager N., Wikler D., De Witte O., Bruneau M., Rorive S., David P., Brotchi J. (2009). Positron Emission Tomography-Guided Volumetric Resection of Supratentorial High-Grade Gliomas: A Survival Analysis in 66 Consecutive Patients. Neurosurgery.

[8] Pöpperl G., Kreth F.W., Mehrkens J.H., Herms J., Seelos K., Koch W., Gildehaus F.J., Kretzschmar H.A., Tonn J.C., Tatsch K. (2007). FET PET for the evaluation of untreated gliomas: Correlation of FET uptake and uptake kinetics with tumour grading. Eur. J. Nucl. Med. Mol. Imaging.

[9] Thon N., Kunz M., Lemke L., Jansen N.L., Eigenbrod S., Kreth S., Lutz J., Egensperger R., Giese A., Herms J. (2014). Dynamic 18F-FET PET in Suspected WHO Grade II Gliomas Defines Distinct Biological Subgroups with Different Clinical Courses. Int. J. Cancer.

[10] Jansen N.L., Suchorska B., Wenter V., Schmid-Tannwald C., Todica A., Eigenbrod S., Niyazi M., Tonn J.C., Bartenstein P., Kreth F.W. (2015). Prognostic Significance of Dynamic 18F-FET PET in Newly Diagnosed Astrocytic High-Grade Glioma. J. Nucl. Med..

[11] Röhrich M., Huang K., Schrimpf D., Albert N.L., Hielscher T., von Deimling A., Schüller U., Dimitrakopoulou-Strauss A., Haberkorn U. (2018). Integrated Analysis of Dynamic FET PET/CT Parameters, Histology, and Methylation Profiling of 44 Gliomas. Eur. J. Nucl. Med. Mol. Imaging.

[12] Kunz M., Albert N.L., Unterrainer M., la Fougère C., Egensperger R., Schüller U., Lutz J., Kreth S., Tonn J.C., Kreth F.W. (2019). Dynamic 18F-FET PET is a Powerful Imaging Biomarker in Gadolinium-Negative Gliomas. Neuro Oncol..

[13] Vettermann F., Suchorska B., Unterrainer M., Nelwan D., Forbrig R., Ruf V., Wenter V., Kreth F.W., Herms J., Bartenstein P. (2019). Non-Invasive Prediction of IDH-Wildtype Genotype in Gliomas Using Dynamic 18F-FET PET. Eur. J. Nucl. Med. Mol. Imaging.

[14] Pyka T., Hiob D., Preibisch C., Gempt J., Wiestler B., Schlegel J., Straube C., Zimmer C. (2018). Diagnosis of Glioma Recurrence Using Multiparametric Dynamic 18F-Fluoroethyl-Tyrosine PET-MRI. Eur. J. Radiol..

[15] Grosu A.L., Astner S.T., Riedel E., Nieder C., Wiedenmann N., Heinemann F., Schwaiger M., Molls M., Wester H.J., Weber W.A. (2011). An Interindividual Comparison of O-(2-[18F]Fluoroethyl)-L-Tyrosine (FET)– and L-[Methyl-11C]Methionine (MET)–PET in Patients With Brain Gliomas and Metastases. Int. J. Radiat. Oncol. Biol. Phys..

[16] Vomacka L., Unterrainer M., Holzgreve A., Mille E., Gosewisch A., Brosch J., Ziegler S., Suchorska B., Kreth F.W., Tonn J.C. (2018). Voxel-Wise Analysis of Dynamic 18F-FET PET: A Novel Approach for Non-Invasive Glioma Characterisation. EJNMMI Res..

[17] Spence A.M., Muzi M., Mankoff D.A., O’Sullivan S.F., Link J.M., Lewellen T.K., Lewellen B., Pham P., Minoshima S., Swanson K. (2004). 18F-FDG PET of Gliomas at Delayed Intervals: Improved Distinction Between Tumor and Normal Gray Matter. J. Nucl. Med..

[18] Debus C., Afshar-Oromieh A., Floca R., Ingrisch M., Knoll M., Debus J., Haberkorn U., Abdollahi A. (2018). Feasibility and Robustness of Dynamic 18F-FET PET Based Tracer Kinetic Models Applied to Patients With Recurrent High-Grade Glioma Prior to Carbon Ion Irradiation. Sci. Rep..

[19] Koopman T., Verburg N., Pouwels P.J.W., Wesseling P., Hoekstra O.S., de Witt Hamer P.C., Lammertsma A.A., Yaqub M., Boellaard R. (2020). Quantitative Parametric Maps of O-(2-[18F]Fluoroethyl)-L-Tyrosine Kinetics in Diffuse Glioma. J. Cereb. Blood Flow Metab..

[20] Pedersen F., Bergströme M., Bengtsson E., Långström B. (1994). Principal Component Analysis of Dynamic Positron Emission Tomography Images. Eur. J. Nucl. Med. Mol. Imaging.

[21] Šámal M., Kárný M., Benali H., Backfrieder W., Todd-Pokropek A., Bergmann H. (1999). Experimental Comparison of Data Transformation Procedures for Analysis of Principal Components. Phys. Med. Biol..

[22] Territo P.R., Riley A.A., McCarthy B.P., Hutchins G.D. (2016). Measurement of Cardiovascular Function Using a Novel View-Sharing PET Reconstruction Method and Tracer Kinetic Analysis. EJNMMI Phys..

[23] Maes F., Collignon A., Vandermeulen D., Marchal G., Suetens P. (1997). Multimodality Image Registration by Maximization of Mutual Information. IEEE Trans. Med. Imaging.

[24] Lowekamp B.C., Chen D.T., Ibáñez L., Blezek D. (2013). The Design of SimpleITK. Front. Neuroinform..

[25] Parker B.J., Feng D. (2005). Graph-Based Mumford-Shah Segmentation of Dynamic PET with Application to Input Function Estimation. IEEE Trans. Nucl. Sci..

[26] Su K.H., Wu L.C., Liu R.S., Wang S.J., Chen J.C. (2005). Quantification Method in [18F]Fluorodeoxyglucose Brain Positron Emission Tomography Using Independent Component Analysis. Nucl. Med. Commun..

[27] Schroeder W., Martin K., Lorensen B. (2010). The Visualization Toolkit.

[28] Yoo T.S., Ackerman M.J., Lorensen W.E., Schroeder W., Chalana V., Aylward S., Metaxas D., Ross W. (2002). Engineering and algorithm design for an image processing API: A technical report on ITK—the Insight Toolkit. Stud. Health Technol. Inform..

[29] Pauleit D., Floeth F., Hamacher K., Riemenschneider M.J., Reifenberger G., Müller H.W., Zilles K., Coenen H.H., Langen K.J. (2005). O-(2-[18F]Fluoroethyl)-L-Tyrosine PET Combined with MRI Improves the Diagnostic Assessment of Cerebral Gliomas. Brain.

[30] Ishiwata K., Kubota K., Murakami M., Kubota R., Sasaki T., Ishil S. (1993). Re-Evaluation of Amino Acid PET Studies: Can the Protein Synthesis Rates in Brain and Tumor Tissues Be Measured In Vivo?. J. Nucl. Med..

[31] Sato K., Kameyama M., Ishiwata K., Hatazawa J., Katakura R., Yoshimoto T. (1992). Dynamic Study of Methionine Uptake in Glioma Using Positron Emission Tomography. Eur. J. Nucl. Med. Mol. Imaging.

[32] Virtanen P., Gommers R., Oliphant T.E., Haberland M., Reddy T., Cournapeau D., Burovski E., Peterson P., Weckesser W., Bright J. (2020). SciPy 1.0: Fundamental Algorithms for Scientific Computing in Python. Nat. Methods.

[33] Feng D., Wong K.P., Wu C.M., Siu W.C. (1997). A Technique for Extracting Physiological Parameters and the Required Input Function Simultaneously from PET Image Measurements: Theory and Simulation Study. IEEE Trans. Inf. Technol. Biomed..

[34] Logan J., Fowler J.S., Volkow N.D., Ding Y.S., Wang G.J., Alexoff D.L. (2001). A Strategy for Removing the Bias in the Graphical Analysis Method. J. Cereb. Blood Flow Metab..

[35] Pedregosa F., Varoquaux G., Gramfort A., Michel V., Thirion B., Grisel O., Blondel M., Prettenhofer P., Weiss R., Dubourg V. (2011). Scikit-learn: Machine Learning in Python. J. Mach. Learn. Res..

[36] Hardee M.E., Zagzag D. (2012). Mechanisms of Glioma-Associated Neovascularization. Am. J. Pathol..

[37] Gunn R.N., Lammertsma A.A., Hume S.P., Cunningham V.J. (1997). Parametric Imaging of Ligand-Receptor Binding in PET Using a Simplified Reference Region Model. NeuroImage.

[38] Panitchob N., Widdows K.L., Crocker I.P., Johnstone E.D., Please C.P., Sibley C.P., Glazier J.D., Lewis R.M., Sengers B.G. (2016). Computational Modelling of Placental Amino Acid Transfer as an Integrated System. Biochim. Biophys. Acta Biomembr..

[39] Okubo S., Zhen H.N., Kawai N., Nishiyama Y., Haba R., Tamiya T. (2010). Correlation of L-Methyl-11C-Methionine (MET) Uptake with L-Type Amino Acid Transporter 1 in Human Gliomas. J. Neurooncol..

[40] Haining Z., Kawai N., Miyake K., Okada M., Okubo S., Zhang X., Fei Z., Tamiya T. (2012). Relation of LAT1/4F2hc Expression with Pathological Grade, Proliferation and Angiogenesis in Human Gliomas. BMC Clin. Pathol..

[41] Moulin-Romsée G., D’Hondt E., de Groot T., Goffin J., Sciot R., Mortelmans L., Menten J., Bormans G., Van Laere K. (2007). Non-Invasive Grading of Brain Tumours Using Dynamic Amino Acid PET Imaging: Does It Work for 11C-Methionine?. Eur. J. Nucl. Med. Mol. Imaging.

[42] Habermeier A., Graf J., Sandhöfer B.F., Boissel J.P., Roesch F., Closs E.I. (2015). System L Amino Acid Transporter LAT1 Accumulates O-(2-Fluoroethyl)-L-Tyrosine (FET). Amino Acids.

[43] Akerele M.I., Karakatsanis N.A., Deidda D., Cal-Gonzalez J., Forsythe R.O., Dweck M.R., Syed M., Newby D.E., Aykroyd R.G., Sourbron S. (2020). Comparison of Correction Techniques for the Spill in Effect in Emission Tomography. IEEE Trans. Radiat. Plasma Med. Sci..

[44] Koopman T., Verburg N., Schuit R.C., Pouwels P.J.W., Wesseling P., Windhorst A.D., Hoekstra O.S., de Witt Hamer P.C., Lammertsma A.A., Boellaard R. (2018). Quantification of O-(2-[18F]Fluoroethyl)-L-Tyrosine Kinetics in Glioma. EJNMMI Res..

[45] Veronese M., Bertoldo A., Tomasi G., Smith C.B., Schmidt K.C. (2018). Impact of Tissue Kinetic Heterogeneity on PET Quantification: Case Study with the L-[1-11C]Leucine PET Method for Cerebral Protein Synthesis Rates. Sci. Rep..

[46] Cavalcanti Y.C., Oberlin T., Dobigeon N., Fevotte C., Stute S., Ribeiro M.J., Tauber C. (2019). Factor Analysis of Dynamic PET Images: Beyond Gaussian Noise. IEEE Trans. Med. Imaging.

[47] Kamenskiy A.V., Pipinos I.I., Carson J.S., MacTaggart J.N., Baxter B.T. (2015). Age and Disease-Related Geometric and Structural Remodeling of the Carotid Artery. J. Vasc. Surg..

